# Single-cell transcriptomic profiling reveals decreased ER protein Reticulon3 drives the progression of renal fibrosis

**DOI:** 10.1186/s43556-024-00187-x

**Published:** 2024-06-28

**Authors:** Shuai Guo, Yi Dong, Ran Du, Yu-Xing Liu, Shu Liu, Qin Wang, Ji-Shi Liu, Hui Xu, Yu-Jie Jiang, Huang Hao, Liang-Liang Fan, Rong Xiang

**Affiliations:** 1 Department of Nephrology, National Clinical Research Center for Geriatric Disorders, Xiangya Hospital, Central South University, Changsha, China; 2 Department of Cell Biology, Hunan Key Laboratory of Medical Genetics, School of Life Sciences, Central South University, Changsha, China; 3 School of Traditional Chinese Medicine, Southern Medical University, Guangzhou, China; 4 Department of Nephrology, The third Xiangya Hospital, Central South University, Changsha, China; 5 Clinical Research Center For Critical Kidney Disease In Hunan Province, Changsha, China; 6 Department of Computer Science, Wake Forest University, Winston-Salem, NC USA; 7 Hunan Key Laboratory of Organ Fibrosis, Central South University, Changsha, China

**Keywords:** Chronic kidney disease (CKD), Reticulon-3, Single-cell transcriptomics, Reactive oxygen species (ROS), Endothelial-to-mesenchymal transition (EndoMT), Cell-cell communication

## Abstract

Chronic kidney disease (CKD) poses a significant global health dilemma, emerging from complex causes. Although our prior research has indicated that a deficiency in Reticulon-3 (RTN3) accelerates renal disease progression, a thorough examination of RTN3 on kidney function and pathology remains underexplored. To address this critical need, we generated *Rtn3*-null mice to study the consequences of RTN3 protein deficiency on CKD. Single-cell transcriptomic analyses were performed on 47,885 cells from the renal cortex of both healthy and *Rtn3*-null mice, enabling us to compare spatial architectures and expression profiles across 14 distinct cell types. Our analysis revealed that RTN3 deficiency leads to significant alterations in the spatial organization and gene expression profiles of renal cells, reflecting CKD pathology. Specifically, RTN3 deficiency was associated with *Lars2* overexpression, which in turn caused mitochondrial dysfunction and increased reactive oxygen species levels. This shift induced a transition in renal epithelial cells from a functional state to a fibrogenic state, thus promoting renal fibrosis. Additionally, RTN3 deficiency was found to drive the endothelial-to-mesenchymal transition process and disrupt cell-cell communication, further exacerbating renal fibrosis. Immunohistochemistry and Western-Blot techniques were used to validate these observations, reinforcing the critical role of RTN3 in CKD pathogenesis. The deficiency of RTN3 protein in CKD leads to profound changes in cellular architecture and molecular profiles. Our work seeks to elevate the understanding of RTN3’s role in CKD’s narrative and position it as a promising therapeutic contender.

**Supplementary Information:**

The online version contains supplementary material available at 10.1186/s43556-024-00187-x.

## Introduction

Chronic kidney disease (CKD) presents a daunting challenge to global health, affecting an estimated 10–15% of the global population [1]. CKD is characterized by a gradual loss of kidney function over time [1]. It is often asymptomatic until reaches an advanced stage. The progression of CKD is characterized by the deposition of a pathological fibrillar matrix in the area between renal tubules and peritubular capillaries. This leads to interstitial fibrosis that disrupts normal tubular function, and subsequently results in diminished kidney volume and compromised perfusion [1–4]. Fibrotic changes also occur in the glomerulus (glomerulosclerosis) and arterioles (arteriolosclerosis), resulting in compromised blood flow and perfusion [4]. Furthermore, fibrosis and fibrogenesis significantly amplify CKD progression, while recent discoveries have shed light on the role of pericytes/perivascular cells in matrix formation in CKD [5]. Besides, a significant portion of CKD cases are associated with genetic factors, with more than 200 candidate genes identified to date [6]. Most of these genes are found to be collagen-related, mitochondria-related, or ion channel-related [6].

The Reticulon-3 (RTN3) protein, a member of the RTN family, is characterized by its signature C-terminal RTN homolog domain (RHD) [7]. RTN3 shapes the structure of the tubular endoplasmic reticulum (ER) via its ω-shaped (wedge-shaped) membrane topology in the N- and C-terminal domains. Our former studies explore RTN3’s role in peripheral human organs. We find elevated RTN3 levels implicate in conditions ranging from obesity to non-alcoholic fatty liver disease, through interacting with the heat shock protein family (Hsp70/HspA5) [8, 9]. Meanwhile, we observed the loss of RTN3 protein phenocopies CKD via activating the *IGF2-JAK2* pathway in proximal tubular epithelial cells [3].

However, the pathology is not confined to only proximal tubule epithelial cells [3]. CKD manifests through dysfunctions in epithelial, endothelial, mesenchymal, and immune cells [2, 4]. As such, understanding RTN3’s intricate crosstalk with each of these cellular components during CKD progression stands paramount. A detailed knowledge of the renal cell atlas under RTN3 deficiency can pave the way not only for a clearer understanding of CKD’s underpinnings but also potential therapeutic breakthroughs.

To address this critical need, in this study, we profile 47,885 cells from the renal cortex region of healthy and *Rtn3*-null mice, with a total of 14 cell types. Our findings indicate that the loss of *Rtn3* markedly alters cellular organization within the renal cortex. We provide an in-depth exploration on the influence of *Rtn3* deficiency on each cell type and the subsequent disruption to cell-cell communication. Using immunohistochemistry and Western-Blot techniques, we validate these observations, thereby reinforcing the pivotal role of RTN3 in CKD pathogenesis. In essence, our work seeks to elevate the understanding of RTN3’s role in CKD’s narrative and position it as a promising therapeutic contender.

## Results

### The expression of Rtn3 is decreased during the CKD progression

**Fig. 1:**
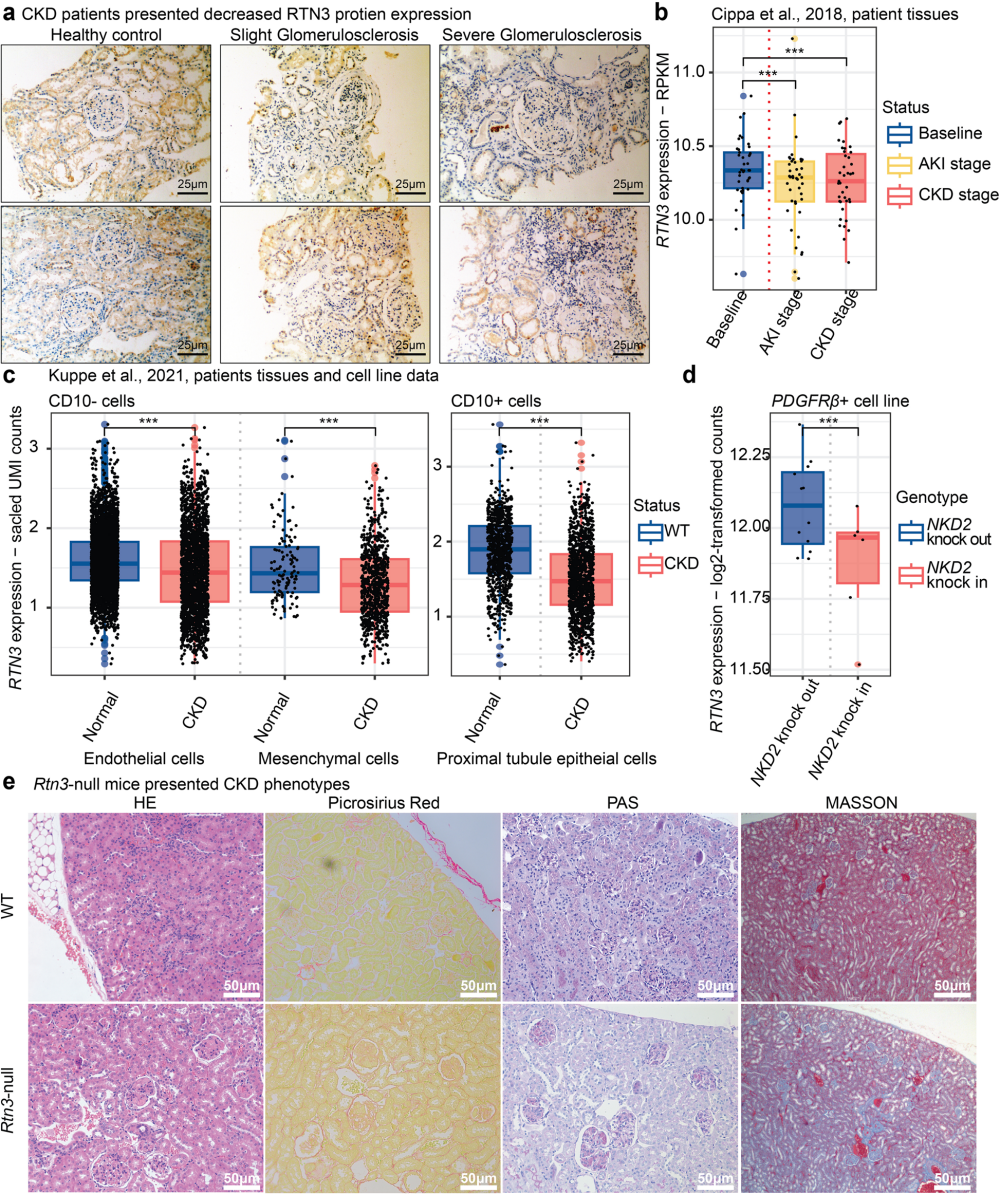
Decreased expression of RTN3 in human CKD conditions. **a** IHC showing RTN3 protein level in human kidney samples in different medical conditions. Left panel, healthy controls (*n* = 2); middle panel, slight glomerulosclerosis (*n* = 2); and right panel, sever glomerulosclerosis (*n* = 2). **b** Box plot illustrating RTN3 expression at different stages in 42 renal transplant recipients. Blue represents baseline stage, yellow indicates AKI stage, and red denotes CKD stage. **c** Box plot showing RTN3 expression between normal and CKD groups across different cell types, including endothelial cells, mesenchymal cells (comprising fibroblasts and myofibroblasts), and proximal tubule epithelial cells (PTs) - blue for normal and red for CKD group. **d** Comparison of RTN3 expression in NKD2 KO and NKD2 OE human cell lines - blue represents the NKD2 KO cell line (rescue group) and red represents the NKD2 OE cell line (CKD model). **e** IHC showing Rtn3 -null mice presenting CKD phenotypes and renal fibrosis. Statistical analyses were conducted using Student’s t-test, with significance determined as *P < 0.05, ** *P* < 0.01, *** *P* < 0.001

To characterize the expression of *RTN3* in CKD kidneys, we performed immunohistochemistry (IHC) staining in two health controls and four CKD patients with different stages (Fig. 1a). Our staining results revealed reduced *RTN3* gene expression during the progression of CKD and renal fibrosis, especially in the renal cortex. To further associate decreased *RTN3* with CKD in human, we analyzed two independent transcriptome datasets [4, 10].

The first dataset was generated containing the bulk mRNA profiling of 42 kidney transplant recipients at different stages [10]. We focused on three stages which included: (1) before implantation, which represented the baseline situation; (2) shortly after the restoration of blood flow in the graft, which represented the acute kidney injury (AKI) condition due to the ischemia reperfusion; and (3) 12 months after transplantation, which represented the CKD condition. Our results showed decreased *RTN3* in both AKI and CKD conditions (Fig. 1b), which were concordant with our previous study [3]. In the second dataset, we characterized the expression of *RTN3* in cell populations from human CKD and normal kidneys in single-cell RNA sequencing (scRNAseq) datasets [4] (Fig. S1). We investigated *RTN3* expression among the CD10- cells (endothelial cells and mesenchymal cells), CD10 + proximal tubule epithelial cells (PTs), and human *PDGFRβ +* cells. The *PDGFRβ +* cells were isolated from healthy human kidney cortex of a nephrectomy specimen [4]. This primary cell line data contained rescue group (*NKD2* knock-out), which represented healthy controls, and a CKD group (*NKD2* knock-in) [4]. We found reduced *RTN3* level among all CKD conditions compared to healthy controls (Fig. 1c, d). Last, we aimed to examine whether the loss of *RTN3* expression was sufficient to induce CKD phenotypes. We generated *Rtn3*-null mice models as previously described [3]. With four distinct staining experiments, we observed pronounced renal injury signals in the *Rtn3*-null mice (Fig. 1e).

In conclusion, our findings emphasize the critical role of *RTN3* loss in renal damage, fibrosis, and the progression of CKD [3, 4, 10]. To further substantiate our proposition that *RTN3* could serve as a therapeutic target for CKD, we propose a more comprehensive investigation in the following sections.

### An overview of the renal cortex cell atlas in healthy and Rtn3-null mice

**Fig. 2:**
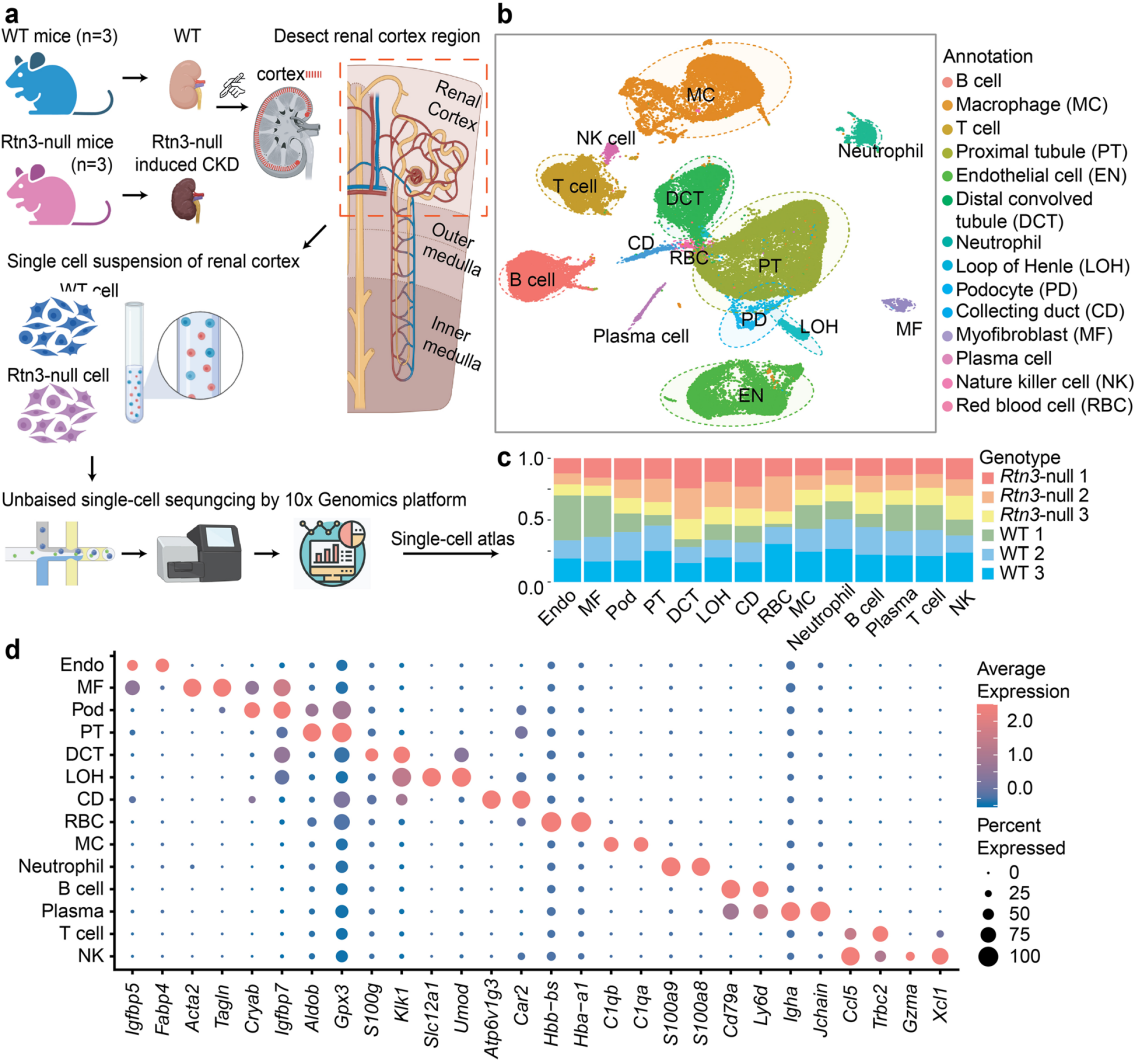
The cell atlas of renal cortex in healthy and ***Rtn3***-null mice. **a** Schematic diagram of the scRNAseq data generation workflow. Three healthy and three *Rtn3*-null renal cortex tissues were surgically isolated. The collected tissue samples were then digested into single cells suspension and sequenced using the 10x Genomics platform. This schematic was created using BioRender.com. **b** UMAP for 14 cell types of 47,885 captured cells in WT and *Rtn3*-null samples after quality controls. **c** Stacked box plot displaying the origin of samples corresponding to each annotated cell type. **d** Dot plot illustrating the expression levels of marker genes for each annotated cell type

We aimed to elucidate the mechanisms underpinning the loss of *RTN3*-induced CKD, focusing on the changes in spatial architectures and molecular profiles. To achieve this, we dissected the renal cortex from three healthy male (WT) mice and three *Rtn3*-null mice. A total of 57,414 cells were isolated and sequenced using the 10x Genomics scRNA-seq platform (Fig. 2a). After quality controls [9] (Fig. S2, Table S1), we included 47,885 cells for annotation and analysis. Graph-based clustering analysis was performed using *Seurat*, yielding 28 distinct cell clusters ranging from as few as 51 cells to as many as 5,783 cells per cluster (Fig. S3a, b). All six kidney samples contributed cells to the unsupervised clusters, with each cluster containing cells from at least four samples (Fig. S3c).

To determine the cell identities, we utilized well-established cell type markers for major kidney cell types from a previous study [2, 11]. A total of 14 cell types were identified (Fig. 2b), including renal tubule epithelial cells (RTECs; which contains proximal tubules, PTs; loop of Henry, LOH; distal convolved tubules, DCTs; and collecting ducts, CDs), podocytes (PDs), immune cells (which contains B cells, T cells, macrophages (MCs), plasma cells, natural killer cells (NKs), and neutrophil cells), red blood cells (RBCs), endothelial cells (ENs), and fibroblasts/myofibroblasts (MFs). Immune and stromal cells were distinct from epithelial cells, while RTECs clusters were closely aligned (Fig. 2b). All major cell types were identified in both WT and *Rtn3*-null mice, indicating unbiased cell-type profiling (Fig. 2c). The top expressed genes for each cell type were presented in Fig. 2d. Most identified genes are cell type markers [11], suggesting a reliable annotation for each cluster.

Lastly, we evaluated the sample origin batch effects and mitochondrial gene content across the six tissue samples. The clustering of cells was not influenced by sample origins or mitochondrial gene contents (Fig. S3d). In comparison with the annotation results (Fig. 2b), we observed that cells with higher mitochondrial mRNAs were enriched in RTECs (Fig. S3e). This observation was consistent with previous studies, which reported those mitochondrial proteins were associated with high expression of solute transport pathways rather than cellular stress responses [11]. Collectively, our single-cell experiments generated a comprehensive cell atlas encompassing 14 previously defined cell types within the renal cortex region (Fig. S3f).

### Rtn3-null changes spatial architectures and molecular profiles in the renal cortex

**Fig. 3:**
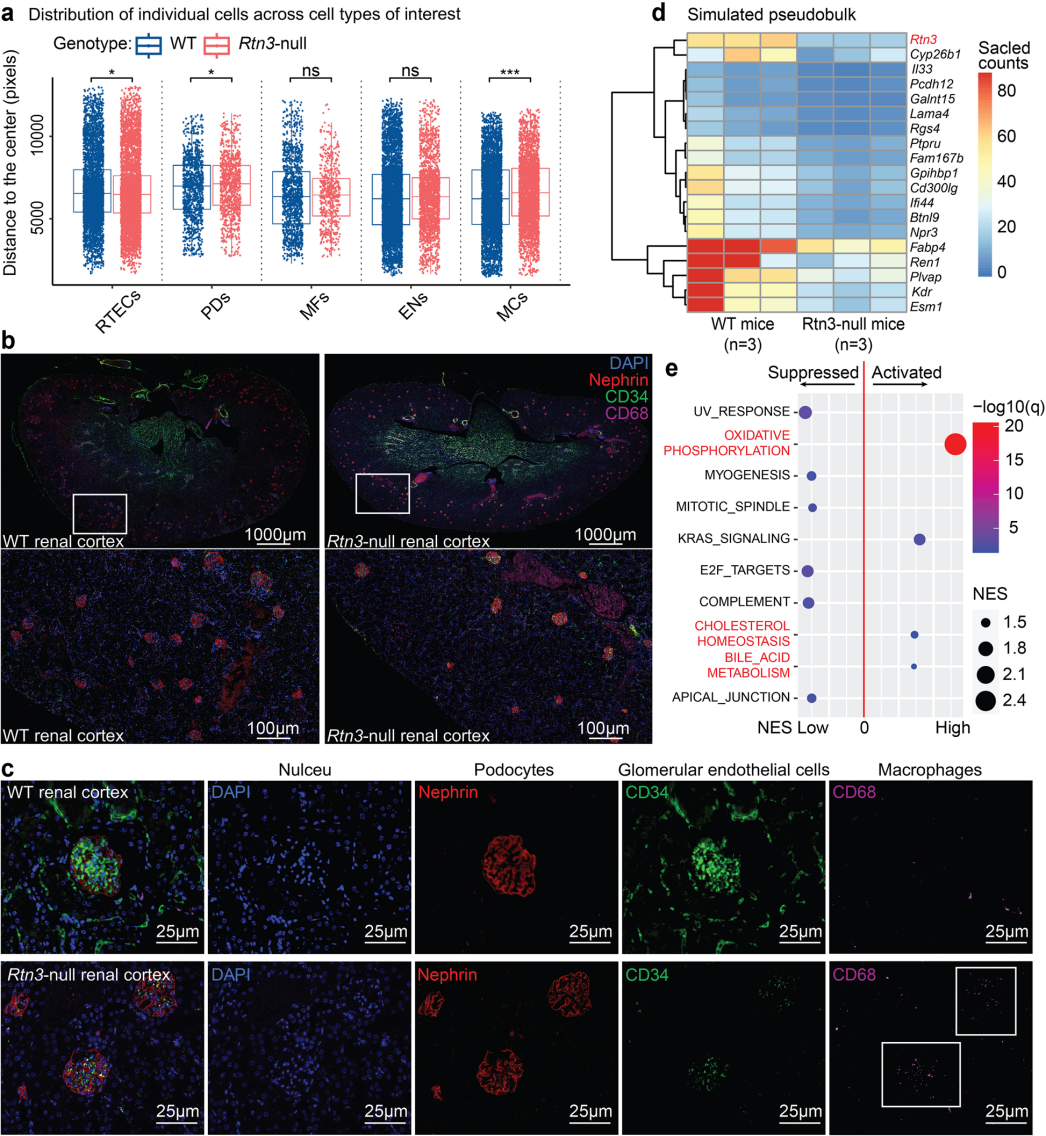
The cellular and molecular architecture of renal cortex in healthy and ***Rtn3***-null mice. **a** Box plots illustrating the spatial distribution of various cell types, estimated via *CellTrek*. Each cell’s distance was determined using Euclidean distance calculations from each spot to the central coordinates. **b** and **c** Multiplex immunohistochemical staining indicating the cellular distribution within the renal cortex of both WT and *Rtn3*-null mice. Blue, DAPI, nuclei; Red, Nephrin, PDs; Green, CD34, ECs; Purple, CD68, MCs. **d** Pseudo-bulk representation simulated by summing up all cells within each scRNAseq data set. **e** GSEA identifying both activated and suppressed pathways in the *Rtn3*-null group. Statistical analyses were conducted using Student’s t-test, with significance determined as *P < 0.05, **P < 0.01, ***P < 0.001. Instances where statistical difference is not significant are marked as *ns*

Previous studies highlighted changes in spatial architectures in CKD and fibrosis kidneys [3]. Our research sought to understand the cellular spatial organization within the renal cortex of *Rtn3*-null models. Using spatial transcriptomics (ST) data from healthy mouse kidneys (sourced from 10x Genomics) as a reference, we segmented the cortex region spots using *Loupe*. We then mapped our sc data to the selected region using *CellTrek* [12] (Fig. S4). We assessed the spatial distribution of cell types of interest by calculating the distance of mapped cells to the tissue center (Fig. 3a). We observed that *Rtn3*-null RTECs localized closer to the renal medulla (BH *p-value* = 0.019), indicating a loss of tubule epithelial cells in the cortex. Contrastingly, the spatial distribution of MFs and ENs appeared largely consistent between both WT and Rtn3-null kidneys (BH p-values of 0.569 and 0.179, respectively). A significant increase of MCs in the cortex was observed under Rtn3-null conditions (BH p-value = 3.16 × 10^−12^).

Immunofluorescence staining results offered some challenges to our mapping predictions, providing new insights into the spatial architectures under *Rtn3*-null (Fig. 3b). Contrary to the predicted increase in PDs within the renal cortex, we noted a decrease in both glomerular number and PDs within individual glomerular structures (Fig. 3c, red). While the general spatial presence of ECs was unaffected by *Rtn3*-null (Fig. 3b, green), we observed a marked decrease in ECs within the glomeruli. The number of infiltrated MCs within the glomeruli notably increased (Fig. 3c, purple), aligning with our predictions.

Shifting our focus to molecular profiles, we simulated six pseudo-bulk mixtures, summing cells from each sample. DE analysis [13] identified significant decreases in *Rgs4* and *Ren1* within the *Rtn3*-null renal cortex (Fig. 3d). Past work has linked *Rgs4*-null mice to increased sensitivity toward renal dysfunction [14]. *Ren1*, a key regulator in the renin–angiotensin–aldosterone system, when reduced, has been associated with nephron-vascular anomalies and striped corticomedullary fibrosis [15]. Our GSEA assessment (Fig. 3e) mirrored prior findings [3, 9], showcasing increased activity in mitochondrial functions following Rtn3 knockout, including oxidative phosphorylation, cholesterol metabolism, and bile acid metabolism. We also detected a reduction of cell proliferation, apical junction, and myogenesis pathways, aligning with Rtn3’s pivotal role in cytoskeleton organization and molecule transportation [16, 17]. Interestingly, the most activated pathways were tied to lipid metabolism, which has been recently implicated in CKD progression [3, 18].

Hence, the loss of *Rtn3* profoundly reshaped both the spatial architectures and the expression profiles across various cell types, mirroring the CKD characteristics. Our observations motivated us to perform a detailed functional analysis for each cell type.

### Rtn3-null induced states transition of renal epithelial cells contributes to renal fibrosis

**Fig. 4:**
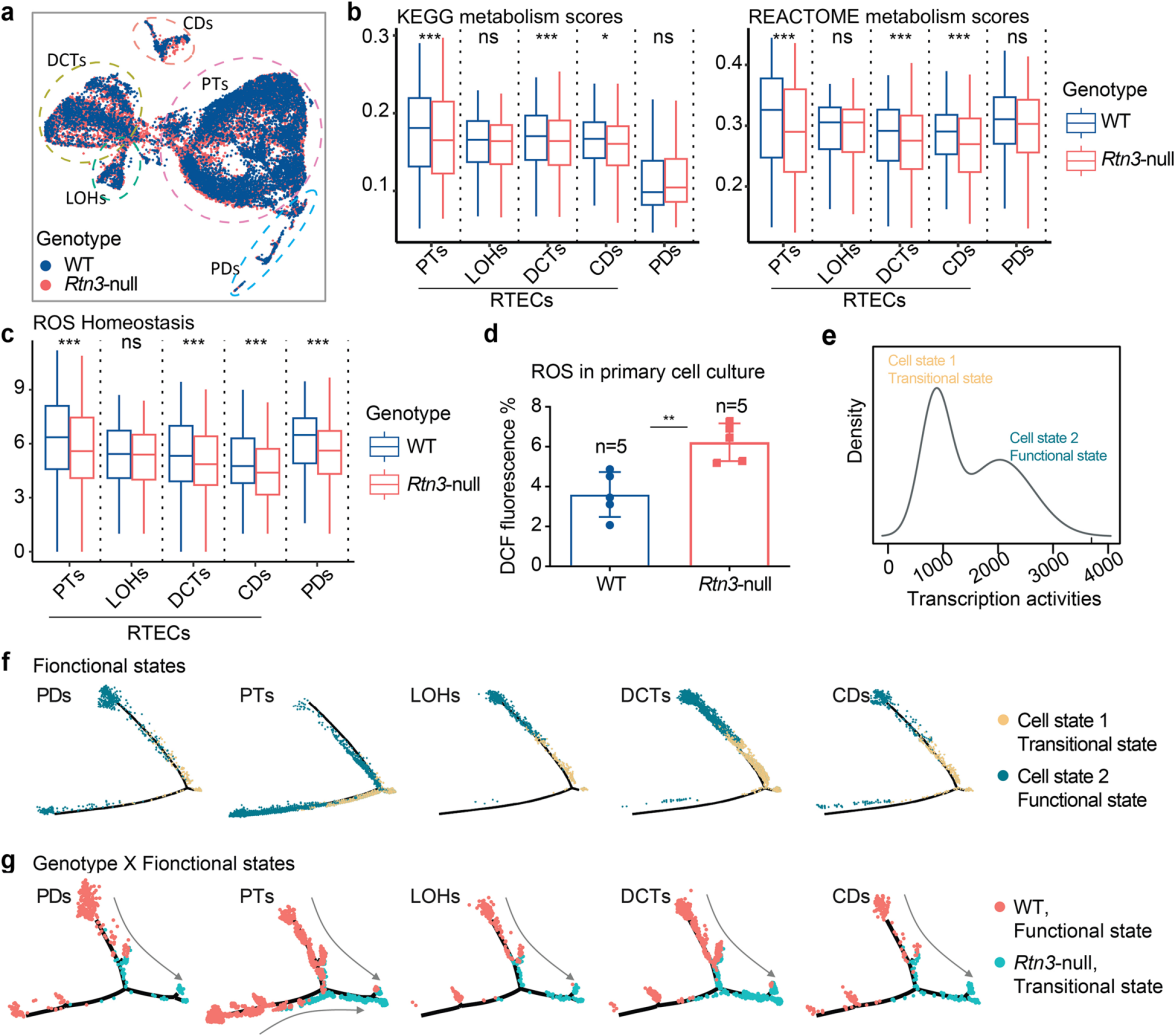
*Rtn3*-null induces states transition of renal epithelial cells through upregulating *Lars2*. **a **UMAP depicting renal epithelial cells (RTECs and PDs) across healthy and *Rtn3*-null samples. **b **Box plots showing the scores of KEGG and REACTOME metabolism between healthy and *Rtn3*-null cells. **c **Box plots presenting the ROS homeostasis scores contrasting healthy and Rtn3-null cells. **d **ROS levels in wild-type control (*n*=5) and *Rtn3*-null primary renal cells (*n*=5). **e **A fitted density plot showcasing the cell transcriptional activities, modelled using a Gaussian finite mixture model with two mixtures. **f **and** g. **Depiction of the lineage relationship in **(f)** functional states and **(g)** genotype X functional states, with arrows indicating potential cell transition directions. Statistical analyses were conducted using Student's t-test, with significance determined as *P< 0.05, **P< 0.01, ***P< 0.001. Instances where statistical difference is not significant are marked as *ns*

We started our analysis with renal epithelial cells. Far from being mere passive elements, renal epithelial cells actively contribute to the disease progression [1, 3, 18]. Considering this, we investigated the effects of *Rtn3*-null on renal cortex epithelial cells, including 18,568 RTECs (PTs, 12,478; LOHs, 782; DCTs, 4,699; CDs, 618) and 670 PDs (Fig. S5a). We compared the molecular signatures of both RTECs and PDs between WT and *Rtn3*-null conditions (Fig. 4a).

We calculated the KEGG and REACT metabolism pathway scores [19] using *scMetabolism* [20] package. The *Rtn3*-null epithelial cells exhibited down-regulated metabolism activities (Fig. 4b). Furthermore, we explored other pathway activities related to CKD, including EMT, inflammation, myogenesis, and TGFβ signaling [21]. In line with our prior investigations, all these pathways demonstrated increased activities in the *Rtn3*-null renal epithelial cells (Fig. S5b). A notable decrease in the ROS homeostasis pathway was evident in the *Rtn3*-null cells (Fig. 4c). To corroborate our observations, we conducted in vitro experiments with *Rtn3*-null primary PTs and then assessed the ROS levels. Compared with the control cell group, the *Rtn3*-null cells exhibited significantly increased ROS (Fig. 4d). Recognizing ROS’s pivotal function in modulating epithelial cell states and rewriting metabolism activities [22], we hypothesized that *Rtn3*-null may enhance the propensity for state transitions in renal epithelial cells.

To validate our hypothesis, we applied a Gaussian finite mixture model (Fig. 4e) to discern potential cell states among all renal epithelial cells, using transcriptional activities as a reliable indicator of cell states [23]. Our analysis revealed two distinct states: the ‘transitional state’ or cell state-1, typified by subdued transcriptional activity, and the ‘functional state’ or cell state-2, distinguished by a heightened transcriptional level (Fig. S5c, d). Functional analysis suggested that the transitional state was characterized by reduced adipogenesis, fatty acid metabolism, and oxidative phosphorylation (Fig. S5e). Conversely, it showed an elevation in EMT, myogenesis, and collagen production, predominantly observed in PDs and PTs (Fig. S5e). We found that WT RTECs were enriched in the functional state, whereas *Rtn3*-null RTECs were enriched in the transitional state (*Chi-squared p-value* < 0.001). Lineage tracing analysis with *Monocle2* [24] confirmed this state-transition process across RTECs and PDs (Fig. 4f). This shift in lineage was more evident upon comparing functional WT epithelial cells with transitional *Rtn3*-null epithelial cells (Fig. 4g). Conclusively, the absence of *Rtn3* in renal epithelial cells amplifies their tendency to shift from a functional to a transitional state. This cellular state transition aligns with prevailing understandings: *Rtn3*-null leads to mitochondrial dysfunction in RTECs without external stimuli [3], and *Rtn3* negatively regulates the intracellular transport [9].

### Lars2 is the molecular driver for Rtn3-null induced states transition in renal epithelial cells

**Fig. 5:**
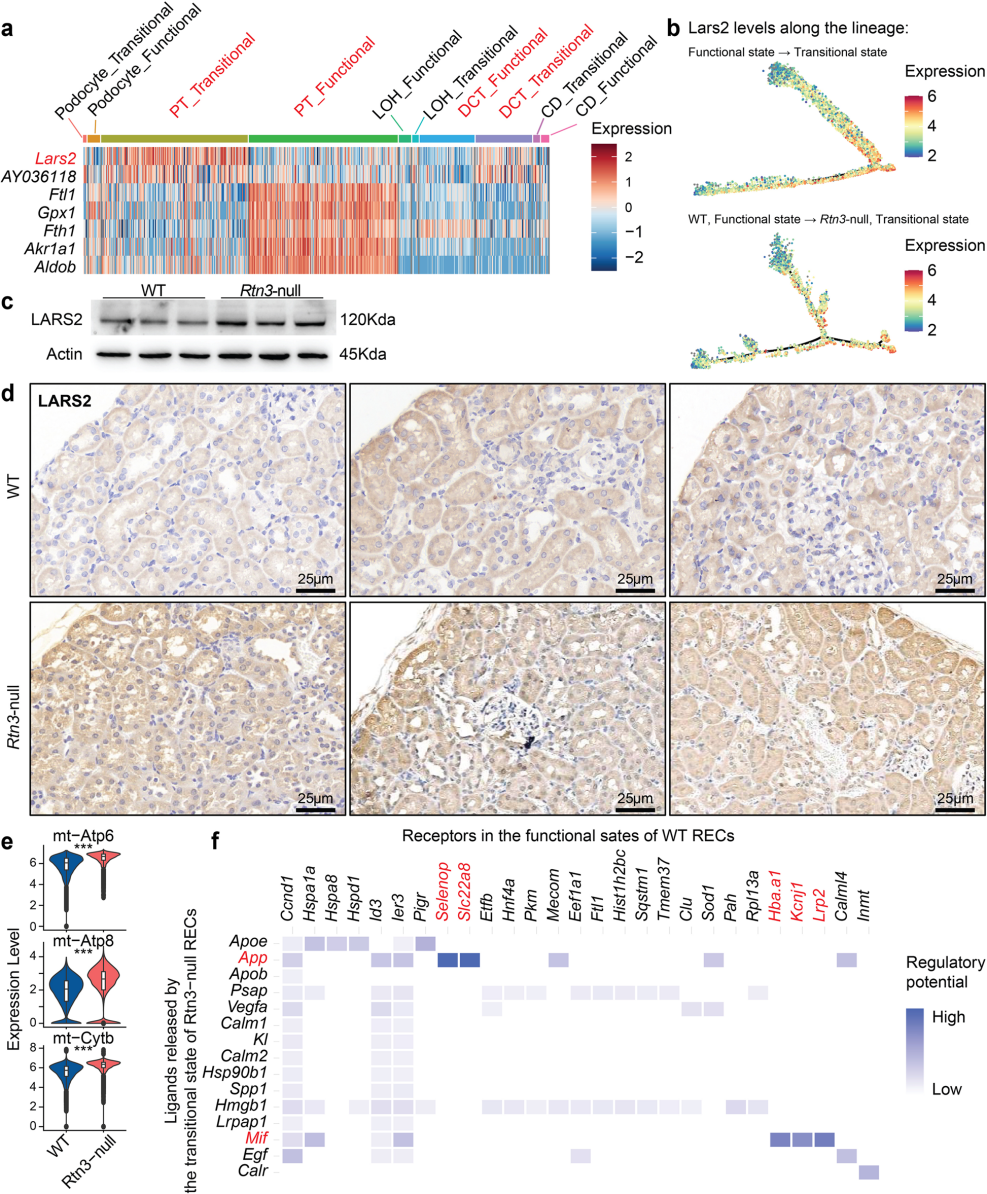
Molecular mechanisms under the cell states transition in renal epithelial cells. **a** A heatmap showcasing the differential gene expression across various cell types between functional and transitional cells. **b **Tracing the expression of *Lars2* along the cell lineage. **c **WB showing the increased Lars2 protein in *Rtn3-null* renal cortex. **d **IHC staining showing the *in-situ* Lars2 protein between WT and *Rtn3*-null renal cortex. **e **Violin plots revealing the expression of mitochondrial genes between WT and *Rtn3*-null cells. **f **A ligand-receptor interaction heatmap between transitional Rtn3-null cells and functional WT cells. Statistical analyses were conducted using Student’s t-test, with significance determined as *P < 0.05, **P < 0.01, ***P < 0.001. Instances where statistical difference is not significant are marked as *ns*

To understand how decreased *Rtn3* drives the cell state transitions in renal epithelial cells remained elusive, we employed a DE analysis across all epithelial cells. Interestingly, *Lars2* emerged prominently, being highly expressed in the transitional clusters (Fig. 5a), especially within PDs, PTs, and DCTs - cell types intricately involved in renal fibrosis [1]. *Lars2*, known for its role as a mitochondrial leucyl-tRNA synthetase, catalyzes the charging of tRNA^Leu(UUR)^ with leucine, a critical step in protein synthesis [25]. This enzyme underpins the production of the mitochondrial complex and thereby the preservation of mitochondrial function. As we traced the cell state lineage, a marked increase in *Lars2* expression became evident (Fig. 5b), suggesting a driving function for *Lars2*. Solidifying this observation, both WB and IHC staining confirmed that in Rtn3-null samples, Lars2 protein levels were significantly elevated (Fig. 5c, d). The overexpressed *Lars2* propels an increase in *Atp6, Apt8, and Cytb* levels (Fig. 5e). Current studies [26, 27] revealed that overexpression of those mitochondrial genes generated increased reactive oxygen species (ROS) accompanied by increased oxygen consumption and lactate production, which further impairs mitochondrial function. We further established the direct association between Lars2 and Rtn3-null-induced CKD. We observed an increased LARS2 in CKD patients at both protein and transcriptome levels, as well as in *Rtn3*-null primary cultured PT cells (Fig. S6). Notably, hypoxic reperfusion further increases LARS2 expression in both WT and *Rtn3*-null PT cells, with a more pronounced upregulation in *Rtn3*-null cells. This implies that the lack of *Rtn3* may sensitize PT cells to hypoxic stress, resulting in a greater induction of LARS2 expression.

The next layer of our inquiry revolved around understanding the potential interaction between the two distinct RTEC states. Using *NicheNet*, we probed inter-state communications (Fig. 5f). Our analysis assigned transitional *Rtn3*-null epithelial cells as the primary source of ligands, while the receptors were the WT functional cells. We identified several ligand-receptor (L/R) pairs, including *App-Selenop/Slc22a8* and *Mif-Hba.a1/Kcnj1/Lrp2*. Both these axes are critical for maintaining the normal function of PTs [28, 29]. This suggested a profound implication: *Rtn3*-null-induced transitional epithelial cells might release ligands that compromise the integrity and function of WT cells, further inhibiting the conventional function of PTs.

Joining these findings into a comprehensive narrative, the picture becomes clearer. The deficiency of *Rtn3* triggers an overexpression of *Lars2*. This overexpression propels an increase in mitochondrial gene levels. A consequence is the rampant accumulation of ROS. Known for its pernicious effects, ROS is a catalyst for renal fibrosis. This surge in ROS levels drives the renal epithelial cells to transition from their natural, functional state into a fibrogenic, transitional one. Interestingly, these fibrogenic-transitional renal epithelial cells do not operate solely. They actively engage with normal renal epithelial cells through signaling pathways, with *App-Selenop/Slc22a8* and *Mif-Hba.a1/Kcnj1/Lrp2* being the key communicators. Such interplay can intensify renal fibrosis, underscoring the multifaceted risks posed by the loss of *Rtn3*.

### Rtn3-null induced endothelial-to-mesenchymal transition contributes to renal fibrosis

Endothelial cells (ENs) are critical in maintaining kidney architecture and function [2]. They are implicated in the progression of CKD via endothelial-to-mesenchymal transition (EndoMT) [30, 31]. EndoMT involves ENs undergoing phenotypic changes into mesenchymal cells marked by altered morphology, loss of adhesion, and heightened invasiveness. Concurrently, there’s a biochemical shift with the decline of endothelial markers and the emergence of mesenchymal markers [30, 31]. Despite its potential therapeutic significance in CKD, EndoMT research is hampered by definitional ambiguities and a limited understanding of its pathological role in CKD.

**Fig. 6:**
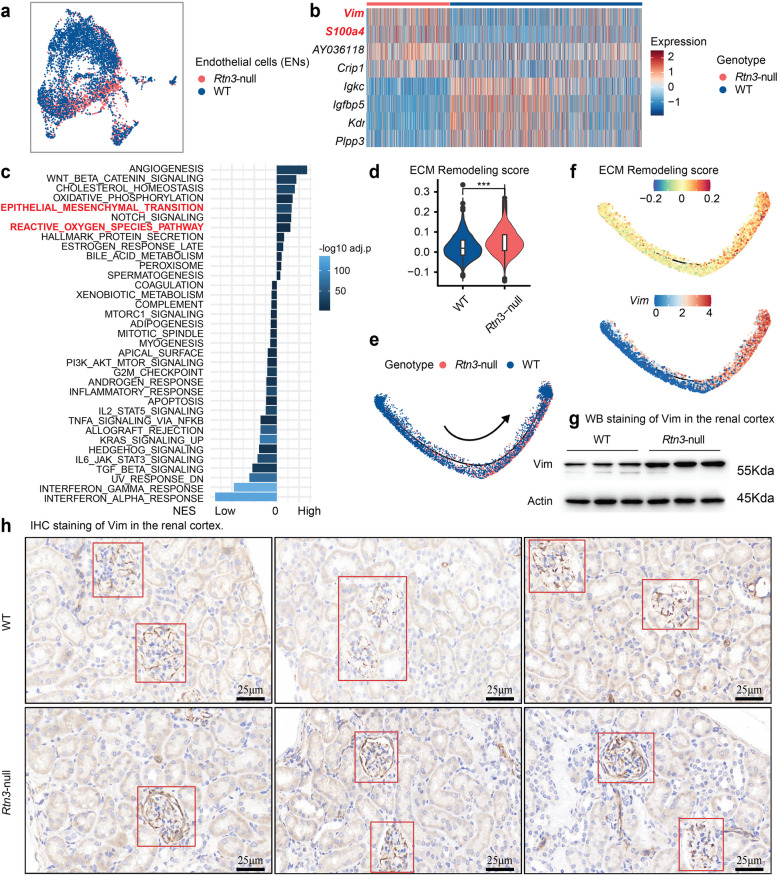
***Rtn3***-null induces endothelial-to-mesenchymal transition. **a** UMAP representation of renal endothelial cells across both healthy and Rtn3-null samples. **b** A heatmap illustrating the differential gene expression between healthy and Rtn3-null endothelial cells. **c** A bar plot reflecting KEGG pathway scores for *Rtn3*-null endothelial cells. **d** A violin plot exhibiting ECM remodeling scores in comparison between healthy and *Rtn3*-null endothelial cells. **e** Predicted lineage relationships between healthy and Rtn3-null endothelial cells, with arrows indicating potential directions. **f** A display of ECM remodeling pathway scores (Top), alongside *Vim* (middle) and *S100a4* (bottom) expression scores mapped along the predicted lineage. **g** and **h** Western blot and IHC validation of *Vim* protein levels in healthy versus *Rtn3*-null endothelial cells. Statistical analyses were conducted using Student’s t-test, with significance determined as *P < 0.05, **P < 0.01, ***P < 0.001

Our previous immunofluorescence results provided a marked decrease in ECs within the glomeruli (Fig. 3c). We postulated that loss of ECs within the glomeruli was driven by *Rtn3*-null induced EndoMT. To validate this, we investigated the effects of *Rtn3*-null on ENs, focusing on associated molecules and EndoMT pathways. We captured 6,087 ENs in our single-cell atlas, with 4,250 and 1,837 cells from WT and *Rtn3*-null mice, respectively (Fig. 6a). Notably, *Rtn3*-null ENs exhibited an increased expression of the fibroblast markers [30, 31] *Vim* and *S100a4* (also known as fibroblast specific protein 1; *Fsp1*) (Fig. 6b). Further, we identified an up-regulation in two EndoMT-related genes in Rtn3-null ENs: *Sox17*, a developmental transcription factor linked to endothelial-to-fibroblast conversion [32], and *Fxyd5*, associated with cystic fibrosis airway epithelia [33].

Pathway analysis indicated that the EMT pathway, reminiscent of fibroblast signatures, was notably active in Rtn3-null ENs (Fig. 6c). Besides, these cells showed heightened ECM remodeling activities (Fig. 6d). Using lineage tracing with *Monocle2*, we discerned a clear lineage from WT to *Rtn3*-null ENs (Fig. 6e), corroborated by escalating EndoMT-associated signals like activated ECM remodeling and rising fibroblast marker gene levels, such as *Vim* (Fig. 6f). The increased Vim protein levels in *Rtn3*-null kidneys were further substantiated through WB and IHC staining (Fig. 6g, h).

In conclusion, our findings underscore the activation of the EndoMT pathway in *Rtn3*-null ENs. We find loss of *Rtn3* in renal tissues could be one of the molecular drivers catalyzing the EndoMT process, further exacerbating renal fibrosis in CKD.

### Rtn3-null induces renal fibrosis by both epithelial mitochondrial dysfunction and EndoMT

Previously, we detailed the impacts of *Rtn3* loss on epithelial cells and ENs in the context of renal fibrosis. We next aimed to study myofibroblasts (MFs), as they were directly responsible for the synthesis and deposition of fibrogenic ECM components during CKD progression [1, 4]. Additionally, epithelial cells known to stimulate MF proliferation and ENs transitioning into myofibroblasts via EndoMT [3, 30, 31]. This motivated us to focus on how loss of *Rtn3* impacted the cell-cell communication between MFs and epithelial/endothelial cells.

**Fig. 7:**
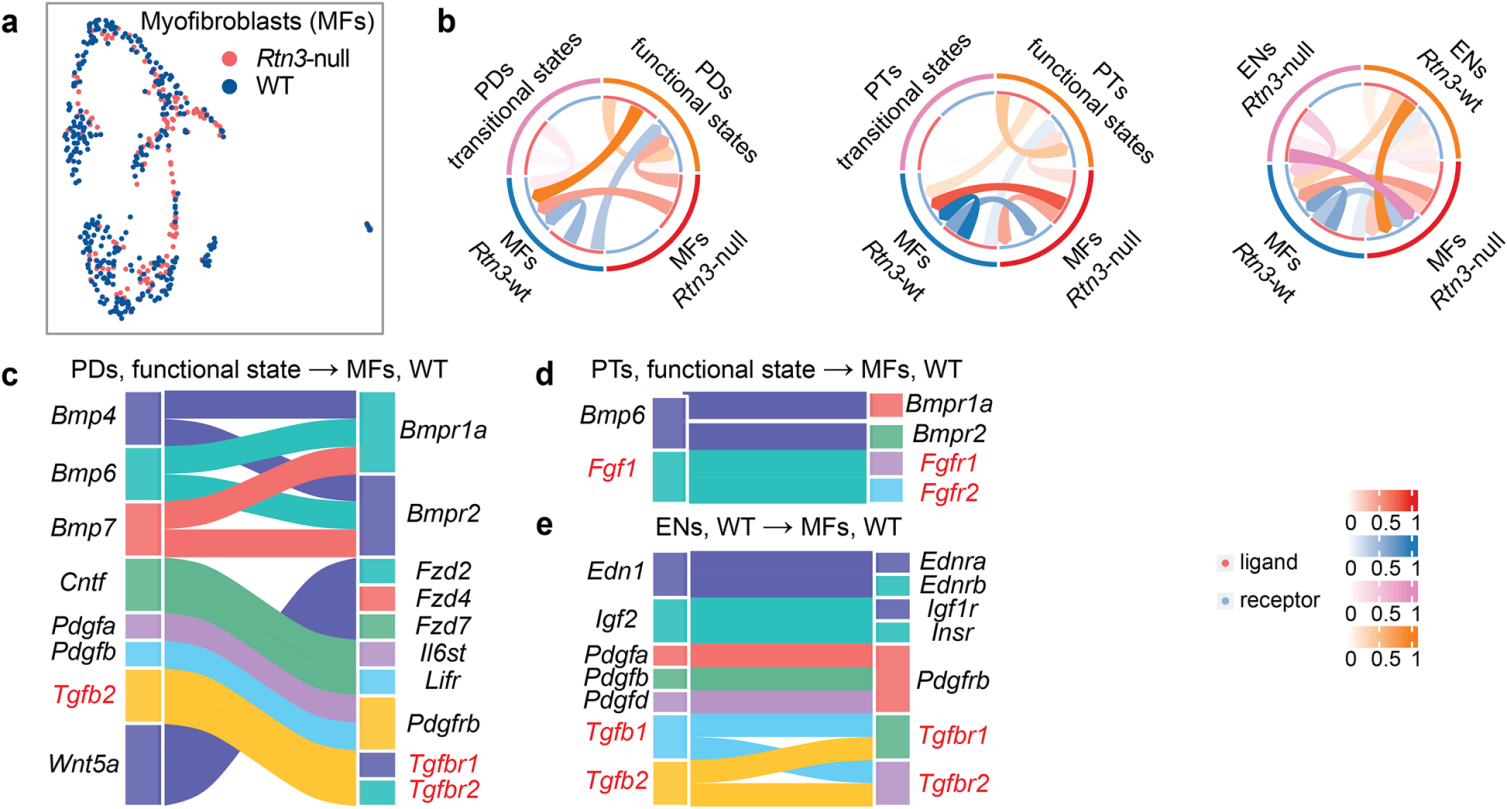
The cell-cell communication of myofibroblasts. **a** UMAP visualization of renal MFs across both healthy and Rtn3-null samples. **b** Cell-cell communication plot exhibiting interactions between PDs and MFs (left), PTs and MFs (middle), and ENs and MFs (right). c, d, and e. Illustrate baseline cell-cell communication pathways under normal conditions: (**c**) Functional PDs to WT MFs; (**d**) Functional PTs to WT MFs; (**e**) WT ENs to WT MFs

We captured 508 MFs (Figs. 7a and 353 cells from WT and *Rtn3*-null mice, respectively). Utilizing *CellChat*, we probed the signaling dynamics between MFs and both epithelial cells and ENs. We focused on PDs and PTs, the two major cell types presented states transition in the *Rtn3*-null model (Fig. 4e). Our findings indicated that *Rtn3*-null led to a pronounced decline in cell-cell interactions, particularly between MFs and transitional PDs/PTs (Fig. 7b). To investigate disrupted cell communications, we established a baseline using functional PDs, PTs, and WT ENs in conjunction with WT MFs (Fig. 7c-e). We revealed prominent axes such as *Tgfb2-Tgfbr1/2* between PDs/ENs and MFs, *Fgf1-Fgfr1/2* between PTs and MFs. Given prior reports linking *Tgfbr2* deletion to enhanced MF recruitment [34], the *Rtn3*-null induced loss of the *Tgfb2-Tgfbr1/2* axis suggests potential hyper-activation of MFs, resulting in renal fibrosis. Delving deeper, our analysis revealed activated TGFβ signaling in *Rtn3*-null PDs and MFs comparing to other pairs (Fig. S7a). The transitional phase of PDs also activated the Jak-Stat pathway in functional PDs, implicating it in CKD progression. Investigating the ENs-MFs dynamic, we observed amplified MAPK pathway signaling under Rtn3-null conditions (Fig. S7b).

In essence, our results provide possible signaling axes important for proper kidney functions. *Rtn3*-null disrupts these axes and potentially exacerbates CKD progression.

### Rtn3-null disturbs the normal immune microenvironment in the renal cortex

Macrophages (MCs) are other key factors leading to renal fibrosis. In our previous section, we observed a significant infiltration of MCs in the glomeruli under *Rtn3*-null condition (Fig. 3a-c). Additionally, previous research suggests that ECs actively recruit MCs to renal sites, thereby exacerbating renal fibrosis progression [35]. Guided by these insights, we aimed to decipher the cell signaling intricacies between ECs and MCs in both WT and *Rtn3*-null settings using *CellChat*.

**Fig. 8:**
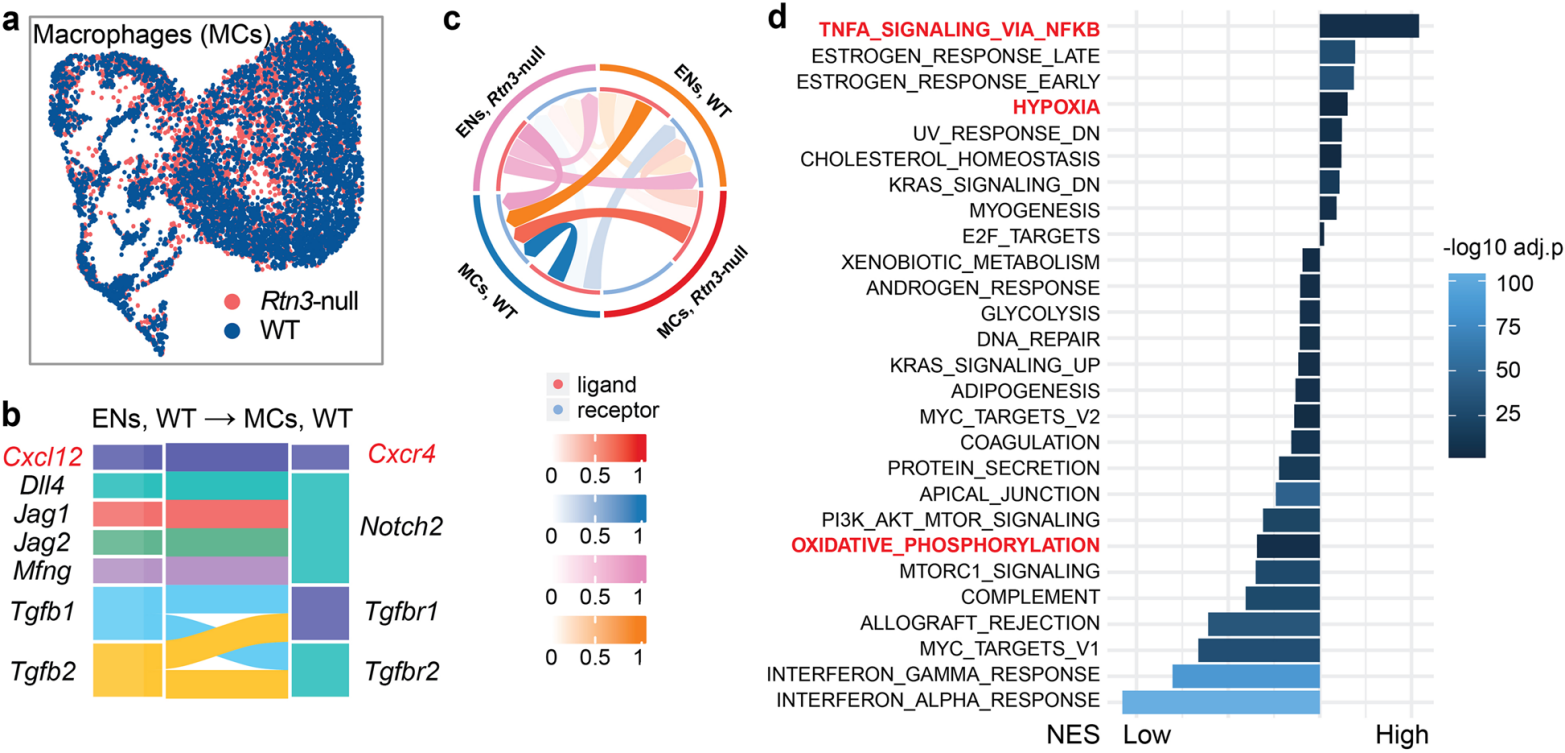
The cell-cell communication between macrophages and endothelial cells. **a** UMAP visualization of renal MCs across both healthy and *Rtn3*-null samples. **b** Cell-cell communication plot exhibiting interactions between ENs and MCs under different genotypes. **c** Illustrate baseline cell-cell communication pathways under normal conditions: WT ENs to WT MCs. **d** A bar plot reflecting KEGG pathway scores for *Rtn3*-null MCs. PDs, Podocytes; PTs, Proximal Tubule Epithelial Cells; ENs, Endothelial Cells; MCs, macrophages; WT, Wild type

Our single-cell atlas captured 9,043 MCs, with 5,604 originating from WT mice and 3,439 from Rtn3-null mice (Fig. 8a). Probing deeper into inter-cellular dialogues (Fig. 8b), we discerned a noticeable disruption in the interaction between *Rtn3*-null MCs and ENs. Predominantly, the baseline communication in WT settings between ENs and ECs pivoted around the *Cxcl12/Cxcr4* axis (Fig. 8c). Within renal confines, *Cxcl12*, a low-weight-molecular chemokines [36], plays a critical role in renal development and modulates kidney pathogenesis by interacting with either *Cxcr4* or *Cxcr7* [36]. Former studies report the capacity of Rtn3 protein to interact with *Cxcr4*, facilitating protein-protein interactions [37]. Thus, loss of *Rtn3* potentially disrupts the *Cxcl12/Cxcr4* axis, leading to compromised cellular communications.

Additionally, the KEGG pathway analysis revealed an increased *TNF-α/NFκB* signaling in the *Rtn3*-null MCs (Fig. 8d). *TNF-α* exerts its pro-fibrotic effect by activating kidney inflammation and renal interstitial cell apoptosis [38]. The augmented *TNF-α* signaling in MCs under *Rtn3*-null conditions rings alarm bells for potential CKD and renal fibrosis progression. Our study also unveiled other shifts: increased hypoxia response and decreased oxidative phosphorylation activity. Both red flags for a chronic inflammation in *Rtn3*-null conditions. Further analysis revealed that, compared to the WT controls, *Rtn3*-null led to the loss of crucial signaling like *Toll* − like receptor signaling, *NOD* − like receptor signaling, and *Notch* signaling (Fig. S7c).

In summation, the loss of *Rtn3* in MCs leads to the disruption of immune homeostasis, resulting in chronic inflammation, which eventually contributes to CDK progression.

## Discussion

Our study shows the critical role of *Rtn3* deficiency during CKD development and progression, whose initial downregulation of *Rtn3* may be attributed to environmental factors or medication usage (e.g., adenine [39]; Fig. S8a). Beginning with the collection of renal cortex samples, we utilize scRNA-seq and profile cellular atlas of renal cortex under *Rtn3*-null conditions. Our DEG analysis across cell types offers insights into the molecular pathways affected by *Rtn3* deficiency. Furthermore, cellular interaction landscape unveils the disrupted intercellular communication with the loss of *Rtn3*. We also performed *in-vitro* assays and histological evaluations to further validate our findings, providing a comprehensive profile of *Rtn3*’s role on CKD progression.

We first co-analyze our sc atlas with a spatial reference [12]. We observe significant alterations in the cellular spatial organization within the *Rtn3*-null renal cortex. Renal epithelial cells are situated closer to the renal medulla while endothelial, mesenchymal, and immune cells were enriched in the outer cortex region. Our observations align with the established knowledge [40]. The loss of glomeruli PDs and ENs is a precursor to glomerulosclerosis and tubular atrophy, whereas interstitial inflammation and fibrosis result from an excess of pro-inflammatory MCs. The molecular lens of our study also revealed unique transcriptomic shifts. We noted an upregulation in pathways related to mitochondrial functions like oxidative phosphorylation, alongside lipid metabolism – both of which are strongly tied to CKD progression [18]. Conversely, we observe decreased cell proliferation, apical junction, and myogenesis signaling pathways. These alterations reflect the pervasive effects of *Rtn3*-null on both the cellular and molecular landscapes of the renal cortex.

We next explore the influence of *Rtn3*-null from the cell-type-specific point of view. Within epithelial cells, *Rtn3* deficiency results in significant alterations in metabolic activities and ROS homeostasis. This disruption, accompanied by increased EMT, inflammation, myogenesis, and TGFβ signaling, implies a potential increase in cell state transitions [4, 22]. ROS drives the cell states transition in the epithelia cells [22]. With *Rtn3* deficiency disrupting ROS homeostasis, we categorize epithelial cells into transitional and functional states. *Rtn3*-null epithelial cells tend to shift from functional state towards the transitional fibrotic state, a critical step in CKD progression. Further investigation shows the cell states transition of renal epithelial cells is driven by Lars2 protein [25, 41].

Our exploration further extended to non-epithelial cells. In ENs, *Rtn3*-null leads to the EndoMT transition marked by increased expression of *Vim* and *S100a4*, contributing to the progression of CKD [30, 31]. The inflammatory response of MCs is activated by *Rtn3* deficiency, thus playing a significant role in CKD progression. Besides cell-specific insights, our study also highlights the overarching impact of *Rtn3*-null on cell-cell communications. Normal regulatory communication axes are disrupted, which potentially perturbs tissue homeostasis and exacerbates disease progression.

In this study, we employed a whole-body knockout model of Rtn3 to investigate its role in CKD progression. This approach allowed us to assess the impact of *Rtn3* deficiency on various cell types and their interactions within the kidney, as well as to examine potential systemic influences on CKD pathogenesis. By studying cell-cell interactions in the context of global *Rtn3* deficiency, we gained valuable insights into the complex mechanisms underlying CKD progression. While we acknowledge the potential limitations of our model, such as the possibility of non-specific effects, we performed validation experiments to ensure the specificity. We measured ROS levels and Lars2 expression in primary cultured PT cells derived from WT and *Rtn3*-null mice (Fig. 5 and Fig. S6). We also show that the observed CKD phenotype is not caused by diabetes or associated ER stress (Fig. S8b). Future studies using kidney-specific knockout models may provide additional insights into the role of Rtn3 during CKD pathogenesis.

In conclusion, our study provides novel insights into the pivotal role of *Rtn3* in CKD. By employing single-cell transcriptomics and functional assays, we have uncovered the profound impact of *Rtn3* deficiency on the cellular states, molecular profiles, and intercellular communication within the renal cortex. Our findings highlight *Rtn3* as a critical regulator of cellular transitions, particularly in renal epithelial and endothelial cells, and identify *Lars2* as a key downstream effector driving mitochondrial dysfunction and renal fibrosis. The significance of our study lies in its potential to guide the development of novel therapeutic strategies targeting *Rtn3* and its associated pathways in CKD management, opening up new avenues for research and clinical intervention.

## Materials and methods

### Human tissues

Human kidney sections were collected from Xiangya Hospital and the Third Xiangya Hospital. This study was conducted in strict accordance with the ethical standards of the Declaration of Helsinki regarding research on human subjects. Additionally, all experimental protocols involving vertebrates or regulated invertebrates were designed and performed in compliance with the Basel Declaration and relevant ethical guidelines to ensure humane treatment and care. The study was approved by the Third Xiangya Hospital of Central South University Ethics Committee (Approval No. 2020-S533, date: September 15, 2020). Informed consent was obtained from all individual participants included in the study.

### Mouse strains, primary cell culture, and key reagents

*RTN3*-null mice were generated, and genotyping was performed as described previously [3, 42]. Specifically, to investigate the role of *Rtn3* in CKD progression, we generated a whole-body knockout mouse model using homologous recombination. The mouse *Rtn3* gene spans approximately 56 kb and consists of 9 exons, with the highly conserved reticulon homology domain (RHD) encoded by exons 4–9. To ensure complete disruption of *Rtn3* function, we designed a targeting construct that removed exons 4–9. The targeting vector was constructed using a 5’ homology arm of ~ 5 kb, which was obtained by restriction enzyme digestion of a bacterial artificial chromosome (BAC) containing the *Rtn3* gene. The 3’ homology arm, spanning 4 kb downstream of exon 9, was cloned into a targeting vector containing a neomycin resistance (*Neo*) cassette for positive selection and a thymidine kinase (TK) cassette for negative selection. The validated targeting vector was microinjected into 129 SvEvTac embryonic stem cells, which were then screened for successful homologous recombination. Correctly targeted embryonic stem cell clones were injected into C57BL/6 blastocysts to generate chimeric mice. Chimeric mice were bred with C57BL/6 mice to obtain heterozygous *Rtn3*-null mice, which were subsequently intercrossed to generate homozygous Rtn3-null mice. The *Rtn3*-null mice were maintained on a congenic C57BL/6 background for subsequent experiments. The WT mice (C57BL/6J) were purchased from Cyagen Company (Suzhou, China). Male mice at 7–8 weeks of age were selected to raise for 12 months to induce renal fibrosis.

Primary proximal tubular epithelial cells were isolated from mouse kidneys as follows [3]: (1) the kidneys were decapsulated and bisected, with the medulla removed; (2) the remaining cortices were finely chopped using a scalpel; (3) the chopped tissue was then digested in 1 mg/mL collagenase type-II at 37℃ for 10 min; (4) the digested kidney tissue was passed through a series of brass sieves with progressively smaller mesh openings; (5) cells were collected from the 40 μm nylon mesh and spun at 150 X g for 10 min; (6) the cell pellet obtained was resuspended in a medium selective for epithelial cell growth; (7) the cells were then seeded onto 1% gelatin-coated tissue culture plates and incubated at 37℃ with 5% CO_2_.

The RTN3 antibody was generated in the Yan laborator [3, 9, 42]. Antibodies against Vim (# 5741) and Actin (# 3700) were purchased from Cell Signaling Technology. Antibodies against LARS2 (17097-1-AP) were purchased from Proteintech Group, Inc.

### Single cells dissociation, cell sorting, and library preparation

Mice kidney tissues were washed directly in Ham’s F12 medium immediately following the surgery. The renal cortex region is surgically dissected from the whole kidney for each sample as previously described [3]. Collected renal cortex tissues were washed for 10 min in Ham’s F12 medium on ice. Renal cortex tissues were enzymatically digested with a dissociation kit (Miltenyi Biotec, 130-095-929) using GentleMACS Dissociator (Miltenyi Biotec, 130-093-235) following the manufacturer’s instructions. The cell suspensions obtained from the GentleMACS Dissociator were applied to 40-mm cell strainer (Corning, 431,750), centrifuge and resuspend the cells in 5mL of red blood cell lysis buffer (ThermoFisher, A1049201) for 5 min, centrifuge and resuspend the cells with ice-cold PBS containing 0.5% BSA.

Cell viability was determined by trypan blue staining with TC20 automated cell counter (Bio-rad, Hercules, CA). The ratio of viable cells in single-cell suspension was required to be more than 85%. Then the concentration of single-cell suspension was adjusted to 700–1,200 cells/mL. The cells were then processed with the Chromium Single Cell 3’ Reagent Kits as the manufacturer’s instruction (v3 chemistry CG000183). The input cells were then loaded onto the channel of Single Cell B Chip (v3 chemistry, PN-1,000,153). The 10x libraries were constructed using Chromium Controller and Chromium Single Cell 3’ Reagent Kits.

### Preprocessing and annotation of human and mice scRNAseq data

The sc library was analyzed with *Seurat* package [43] following the standard pipeline (Fig. S2 and Table. S1). We obtained 57,414 cells in the raw scRNAseq data from six mice models (WT, *n* = 3; *Rtn3*-null, *n* = 3). Based on the distribution of sequencing statistic, including No. of expressed genes, No. of total UMI counts, and the percentage of mitochondrial genes, we set the cut of as: 200 < nFeature_RNA < 4000, nCount_RNA < 40,000, and percent.mt < 65. Cells passed these cutoffs were valid cells and used for downstream functional analysis. As a result, 9,529 cells (16.60%) have been filled, leaving 47,885 cells in the final sc atlas.

### Pseudotime trajectory analysis

The cellular differentiation trajectory was built using the *Monocle2* package [24] (version 2.20.0). Differential expression analysis was performed using the DifferentGeneTest function, and the top 2000 significant genes were chosen to define cell progression. To reduce the dimensions, the DDRTree method was applied. Finally, the pseudotime trajectory was visualized using the plot_cell_trajectory function within the Monocle 2 package.

### Cell-cell communication analysis

To investigate the interactions between different cell types, cell‒cell communication analysis was conducted using the *CellChat* package [44] (version 1.4.0). The resulting signaling pathways were visualized using the netAnalysis_signalingRole_scatter function.

### Immunohistochemistry (IHC) and immunofluorescence staining

IHC was performed to verify the *in-situ* protein expression. Paraformaldehyde-fixed kidney tissues from mice and human were embedded in paraffin and then cut into 6-µm sections. These sections were prepared for staining with an immunohistochemistry Kit and examined by routine light microscopy.

Immunofluorescence staining was conducted to verify the co-expression of target proteins in mice kidney. Sections were blocked and permeabilized with 2% goat serum and incubated with primary antibodies (CD68, CD34 and Nephrin) overnight at 37℃. Fluorescently labeled secondary antibodies were applied the next day. Nuclei were stained with DAPI.

### Western blotting

The kidney tissues were dissociated on ice in radio-immunoprecipitation assay (RIPA) lysis buffer containing protease inhibitor cocktail, and the mixture was incubated for 1 h. The homogenates were then centrifuged at 13,000 g for 90 min at 4 °C to separate the supernatants. Protein concentrations were determined using bicinchoninic acid protein assays and analysis kits. A total of 30 µg of protein lysates were loaded and separated on a 4–12% SDS-PAGE Bis-Tris gel using standard gel electrophoresis methods. The primary and secondary antibodies mentioned earlier were used for the immunoblotting process. Finally, the protein bands were detected using an iBright Gel imaging system from Thermo Fisher Scientific.

### ROS measurement and calculation

Reactive oxygen species (ROS) levels were measured using the DCFH-DA Cellular ROS Detection Assay Kit (Solarbio, CA1410) according to the manufacturer’s instructions. Briefly, primary cultured proximal tubule (PT) cells derived from wild-type (WT) and Rtn3-null mice were seeded in a 96-well plate at a density of 2 × 104 cells per well and allowed to attach overnight. Cells were then washed with 1X buffer and incubated with DCFH-DA working buffer for 20 min at 37 °C in the dark. After incubation, cells were washed with 1X buffer, and fluorescence intensity was measured using a fluorescence microplate reader (Tecan Infinite M200 PRO) with excitation and emission wavelengths of 485 nm and 525 nm, respectively.

ROS levels were calculated by subtracting the fluorescence intensity of the blank wells (containing only 1X buffer) from the fluorescence intensity of each sample well. The corrected fluorescence intensity values were then normalized to the WT control group to obtain relative ROS levels. Five independent experiments (*n* = 5) were conducted, with each group (WT and Rtn3-null) measured in triplicate within each experiment.

Data were presented as mean ± standard deviation (SD) of the five independent experiments. Statistical analysis was performed using a two-tailed Student’s t-test to compare ROS levels between WT and Rtn3-null PT cells. A p-value of less than 0.05 was considered statistically significant.

### Blood glucose test

Eight-week-old male mice were selected for the study. The mice were fasted for 6 h before the induction of diabetes using streptozotocin (STZ). STZ was administered intraperitoneally at a dose of 50 mg/kg body weight for 5 consecutive days. Fasting blood glucose levels were measured 6 h after the last STZ injection and for a total of 12 days. Blood samples were collected from the tail vein. The blood glucose levels were measured using a standard glucometer (Changsha Sinocare Inc.).

### Statistical analysis

The cell states were identified using Gaussian mixture models implemented in the *mclust* packages (version 5.2). We fit a model with G = 2 components to our sc data, using the normalized total mRNA expression as the input. We set the model parameter to be “V” allowing the variances to be unequal (model="V”). The fitted results were presented in Fig. 4e.

Marker genes (or DEGs between two groups) were identified using the *Seurat* FindAllMarkers (or FindMarkers) function. The student’s t-test was used for two-group comparisons. Chi-square test was used for two-by-two contingency tables (Fig. S5d). Pathway enrichment analysis was conducted using the hypergeometric test, and P values were adjusted using the Benjamini-Hochberg (BH) procedure. Significance levels were denoted as follows: *P < 0.05, **P < 0.01, ***P < 0.001, *ns* means not significant.

We collected human transcriptomics data from two independent datasets [4, 10].

The bulk mRNA profiling of 42 kidney transplant recipients at different stages was obtained from GSE126805.

The processed CD10- cells, CD10 + cells, and human *PDGFRβ +* cells data were obtained from the Zenodo data archive (https://zenodo.org/record/405931510.5281/zenodo.4059315).

### Supplementary Information

Supplementary Material 1.

Supplementary Material 2.

## Data Availability

Our processed scRNAseq datasets and the scripts for data analyzing are available at Mendeley data archive (DOI:10.17632/2g6wbhs6d8.1) and GitHub Repository (https://github.com/alfredsguo/rtn3_null_ckd_project).

## Abbreviations

CD: Collecting ducts
CKD: Chronic kidney disease
DCTs: Distal convolved tubules
EndoMT: Endothelial-to-mesenchymal transition
ENs: Endothelial cells
ER: Endoplasmic reticulum
GSEA: Gene Set Enrichment Analysis
IHC: Immunohistochemistry
Lars2: Leucyl-TRNA Synthetase 2, Mitochondrial
LOH: Loop of Henry
MCs: Macrophages
MFs: Fibroblasts/myofibroblasts
NKs: Natural killer cells
PDs: Podocytes
PTs: Proximal tubule epithelial cells
RBCs: Red blood cells
REN1: Renin 1
RGS4: Regulator of G Protein Signalling 4
RHD: RTN homolog domain
ROS: Reactive Oxygen Species
RTECs: Renal tubule epithelial cells
RTN3/Rtn3: Reticulon-3
scRNAseq: Single-cell RNA Sequencing
ST: Spatial transcriptomics
WT: Wild-type
