# Supplementary material for: Single-cell transcriptomic profiling reveals decreased ER protein Reticulon3 drives the progression of renal fibrosis
Source: Mol Biomed. 2024 Jun 28;5:24. doi: 10.1186/s43556-024-00187-x (PMC11211315; doi:10.1186/s43556-024-00187-x)
Supplement: Supplementary file 2 — Supplementary Material 2. [file 43556_2024_187_MOESM2_ESM.docx]

**Supplementary Materials of “Single-cell transcriptomic profiling reveals decreased ER Protein Reticulon3 drives the progression of renal fibrosis”.**

Shuai Guo ^1, 2, *^, Yi Dong ^2, *^, Ran Du ^2^, Yu-Xing Liu ^1^, Shu Liu ^3^, Qin Wang ^1^, Ji-Shi Liu ^4, 5^, Hui Xu ^1^, Yu-Jie Jiang ^2, 6^, Huang Hao ^1, 7, #^, Liang-Liang Fan ^1, 2, #^, Rong Xiang ^1, 2, 7, #^

1. Department of Nephrology, National Clinical Research Center for Geriatric Disorders, Xiangya Hospital, Central South University, Changsha, China.

2. Department of Cell Biology, Hunan Key Laboratory of Medical Genetics, School of Life Sciences, Central South University, Changsha, China.

3. School of Traditional Chinese Medicine, Southern Medical University, Guangzhou, China.

4. Department of Nephrology, the third Xiangya Hospital, Central South University, Changsha, China.

5. Clinical Research Center For Critical Kidney Disease In Hunan Province，Changsha, China.

6. Department of Computer Science, Wake Forest University, Winston-Salem, NC, United States.

7. Hunan Key Laboratory of Organ Fibrosis, Central South University, Changsha, China.

*. These authors contributed equally.

# Corresponding author.

[xyskhuanghao@csu.edu.cn](mailto:xyskhuanghao@csu.edu.cn) (H. Huang),

[swfanliangliang@csu.edu.cn](mailto:swfanliangliang@csu.edu.cn) (L-L. Fan),

[shirlesmile@csu.edu.cn](mailto:shirlesmile@csu.edu.cn) (R. Xiang)

**Supplementary Table 1. Basic statistics of single cell RNA sequencing**

| Sample ID | No. of cells  (before QC) | No. of cells  (after QC) | Median total  UMI counts  per cell | Median gene  counts per cell | Median  Mitochondrial  percentage (%) |
| --- | --- | --- | --- | --- | --- |
| RTN3-WT-1 | 9160 | 7692 | 3986 | 1117 | 8.22 |
| RTN3-WT-2 | 10663 | 8905 | 4024 | 976 | 12.7 |
| RTN3-WT-3 | 12135 | 10558 | 3843 | 914 | 21.07 |
| RTN3-KO-1 | 9490 | 7469 | 3322 | 693 | 35.7 |
| RTN3-KO-2 | 9017 | 7164 | 3715 | 679 | 38.67 |
| RTN3-KO-3 | 6949 | 6097 | 3876 | 983 | 18.36 |
| Sum | 57414 | 47885 |  |  |  |


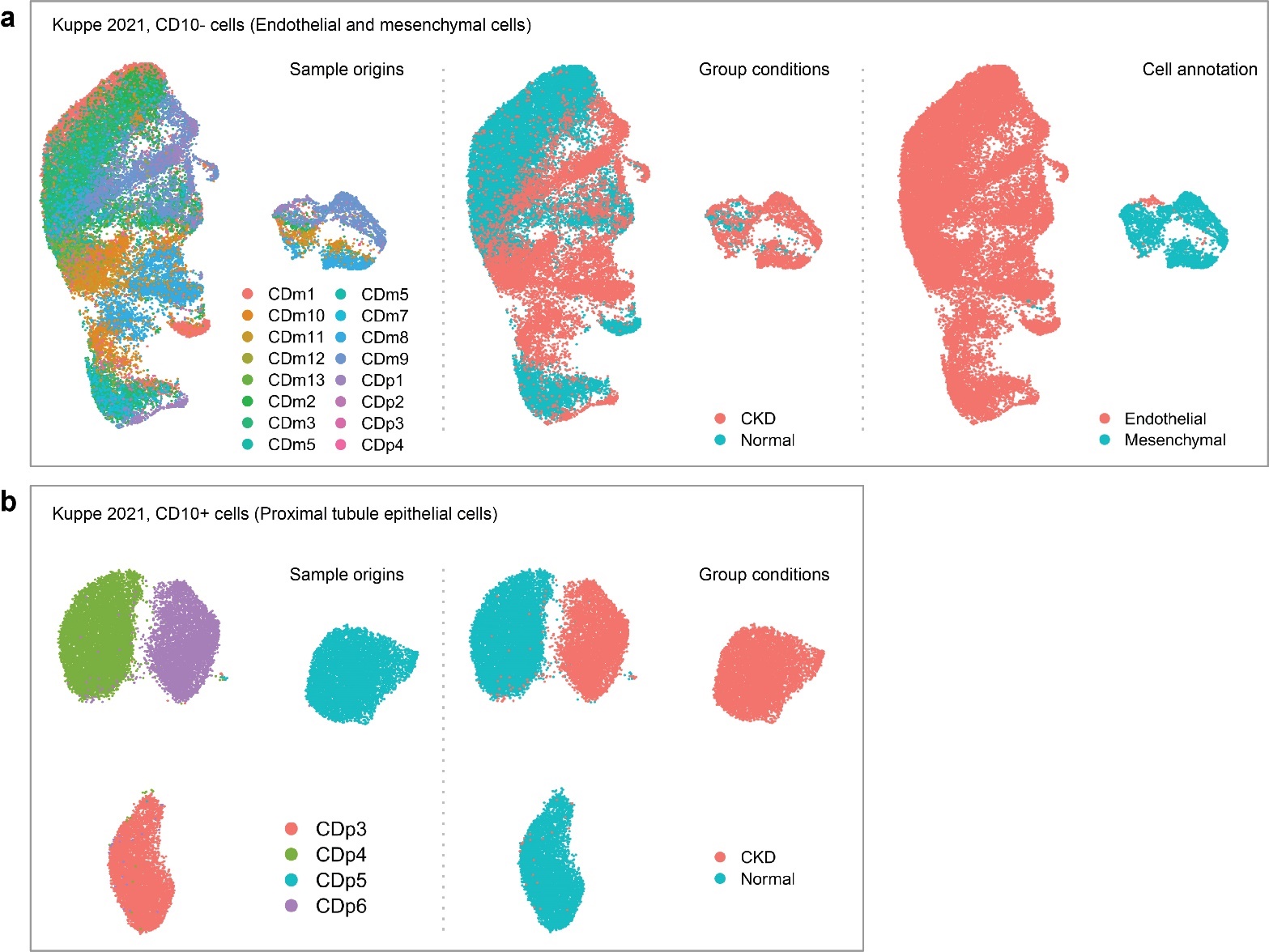


**Supplementary Figure 1. Decreased expression of *Rtn3* in public scRNAseq data.**

**a.** UMAP presenting the scRNAseq data of *CD10*- cells (endothelial and mesenchymal cells) from the *Kuppe et al.*, 2021 study. The left panel depicts the sample origins of the 16 samples, the middle panel indicates group conditions, and the right panel provides cell type annotations. **b.** UMAP showing the scRNAseq data of *CD10*+ cells (proximal tubule cells or PTs) from the same *Kuppe et al.*, 2021 study. Here, the left panel shows the sample origins of the four samples, while the right panel outlines group conditions.


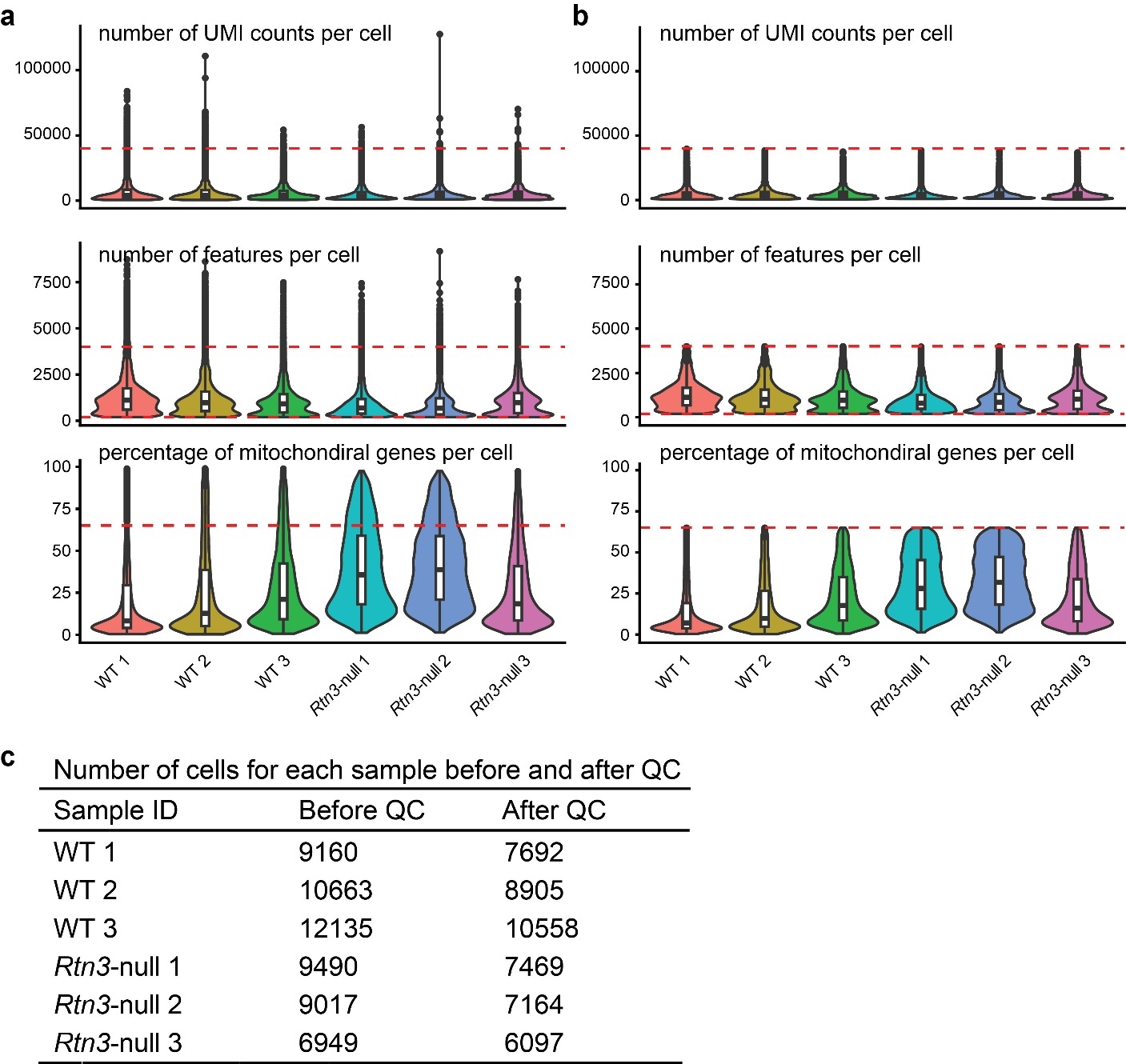


**Supplementary Figure 2. Quality control of renal cortex cell atlas.**

Violin plots showing the sequencing statistics of scRNAseq data before **a** and after **b** quality control steps. **c.** Table containing the number of cells before and after quality control.


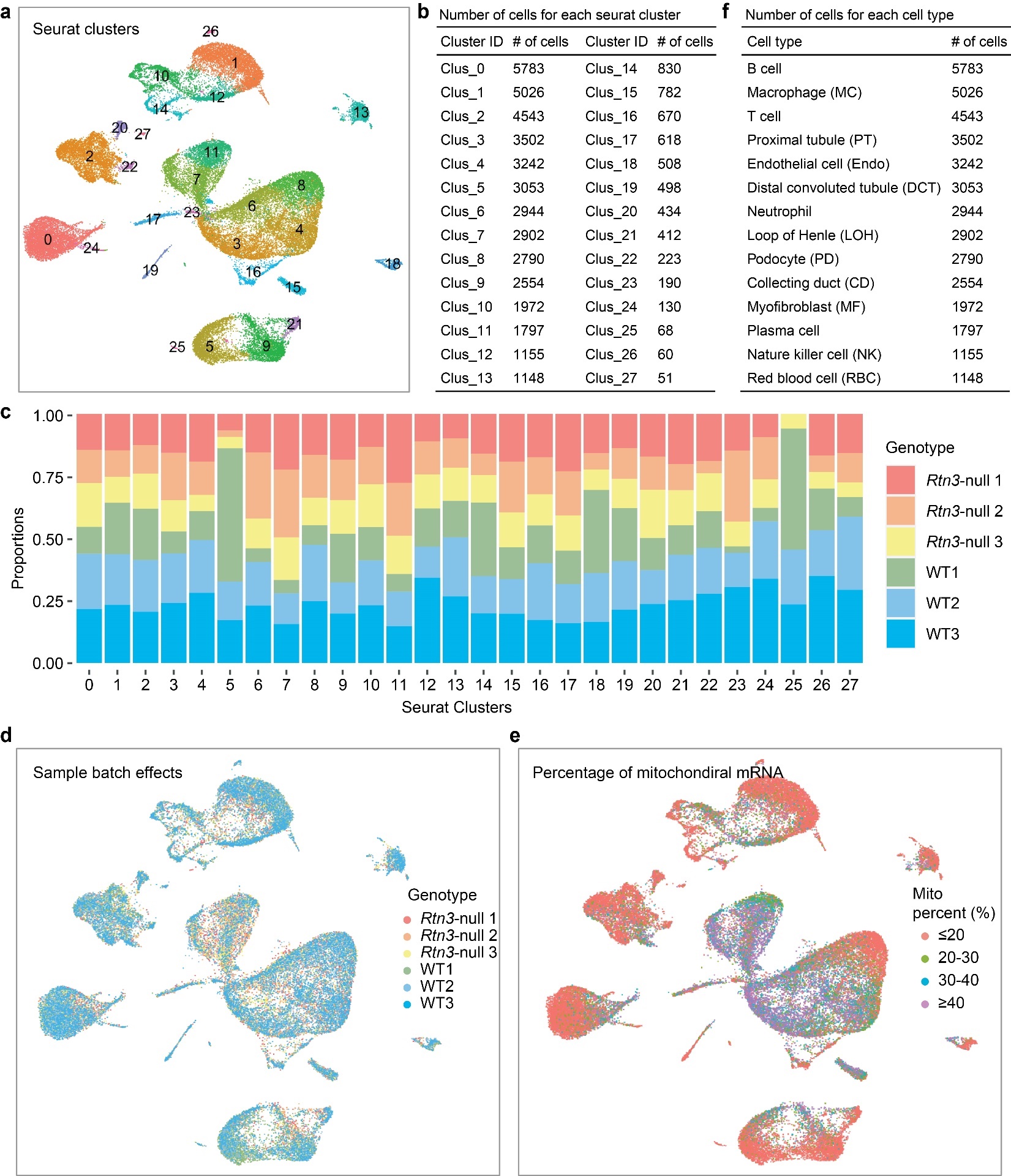


**Supplementary Figure 3. Cell type annotations of renal cortex cell atlas.**

**a.** UMAP representing the results from graphical based clustering results implemented in Seurat package. **b.** Table enumerating the number of cells in each Seurat cluster. **c.** An illustration of sample origins corresponding to each Seurat clustering result. **d.** UMAP visualization highlighting the batch effects on sample origins across the renal cortex tissues of six mice. **e.** UMAP representation illustrating the mitochondrial mRNA percentage distribution across cells. **f.** Table displaying the count of each annotated cell type.


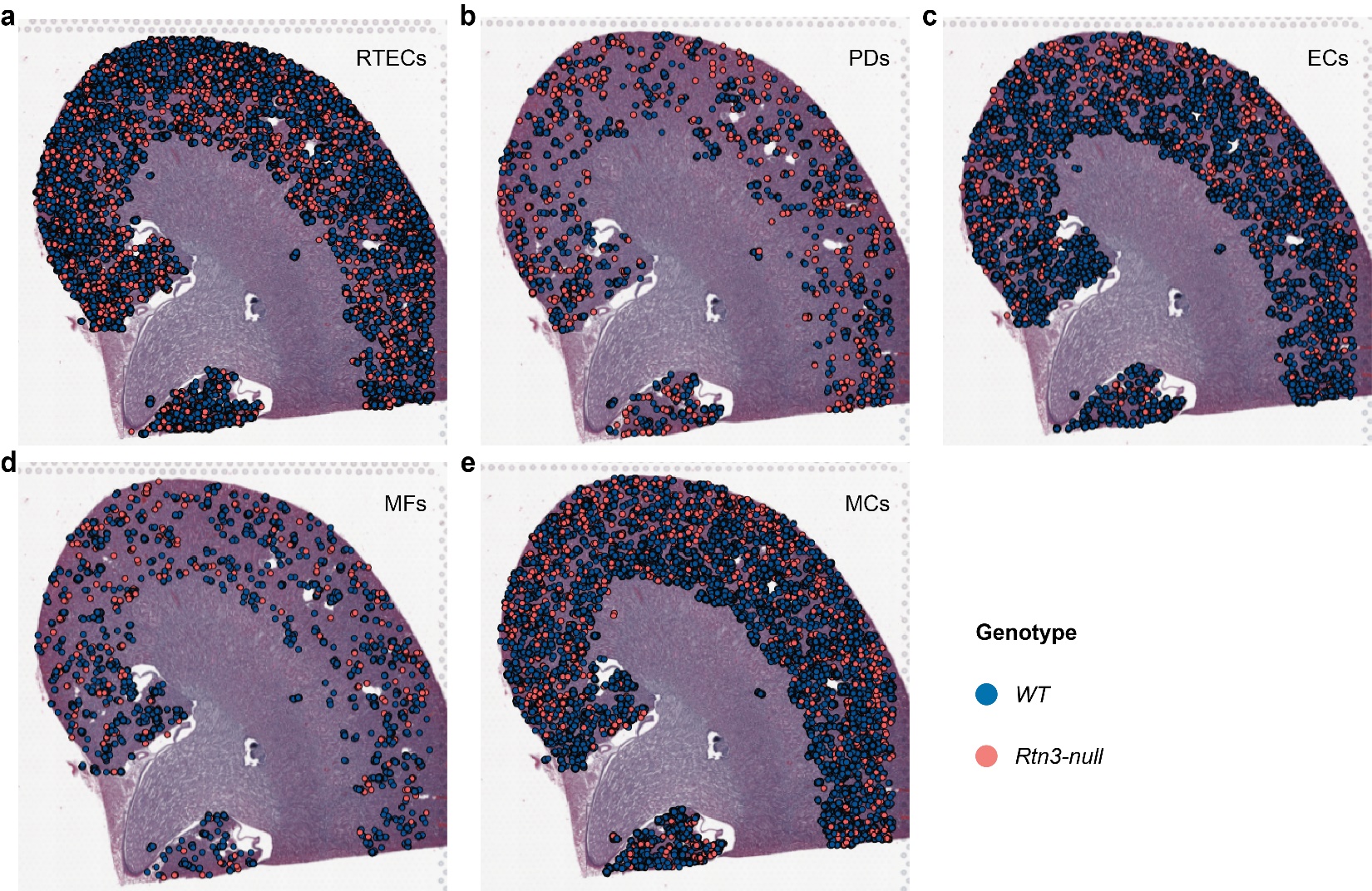


**Supplementary Figure 4. Mapping single-cell to the spatial-resolved transcriptomic data.**

Mapping the cell type of interests to the reference spatial transcriptomics data. **a**, RTECs, renal tubule epithelial cells; **b**, PDs, podocytes; **c**, ECs, endothelial cells; **d**, MFs, fibroblasts/myofibroblasts; **e**, MCs, macrophages.


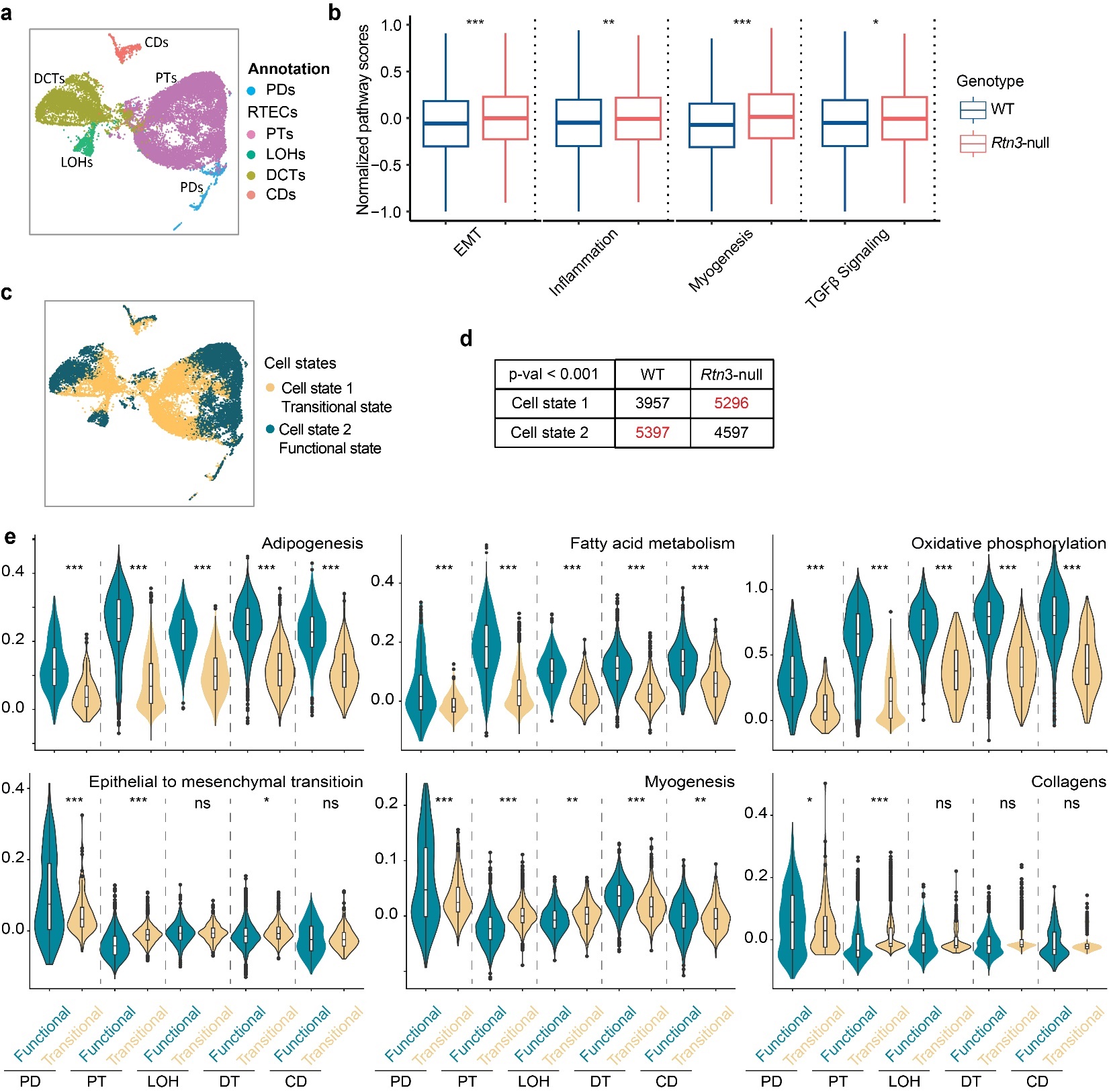


**Supplementary Figure 5. Identification of two cell states among renal epithelial cells.**

**a.** A UMAP representation of renal epithelial cells (RTECs and PDs) annotation. **b.** Box plots illustrating the differences in fibrogenic molecular pathways between healthy and *Rtn3*-null cells. **c.** A UMAP reflecting the different cell states. **d.** A contingency table delineating the correlation between cell states and genotypes among the renal epithelial cells, with a significant *Chi-squared* test result. **e.** Violin plots presenting the scores of metabolic and fibrogenic pathways across the two identified cell states. Statistical analyses were conducted using Student's t-test, with significance determined as *P < 0.05, **P < 0.01, ***P < 0.001. Instances where statistical difference is not significant are marked as *ns*.


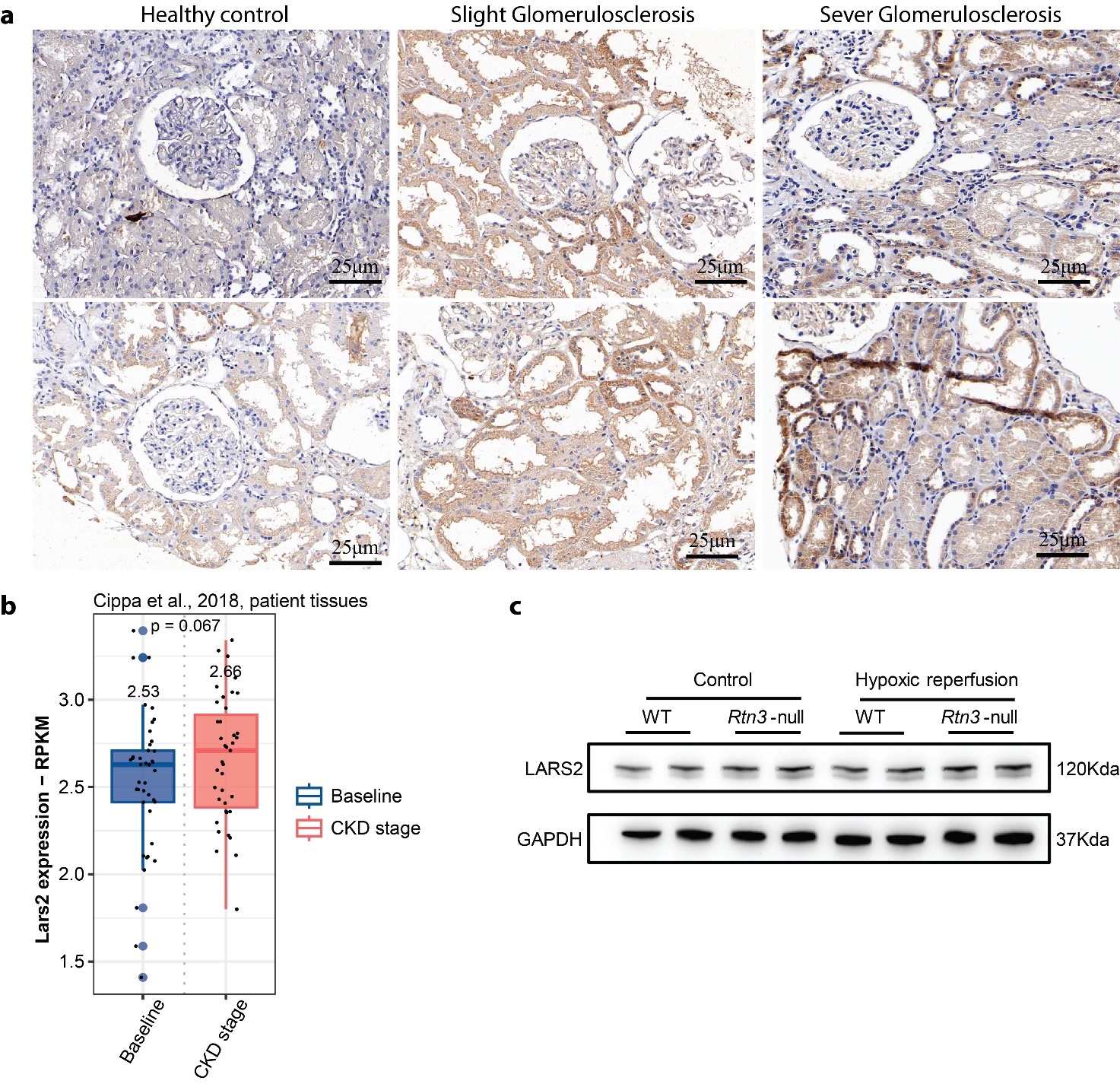


**Supplementary Figure 6. Association of LARS2 and CKD.**

**a.** IHC staining of LARS2 in healthy controls and CKD patients with different disease stages. The protein level of LARS2 is increased with the disease progression. **b.** Expression of *Lars2* mRNA in healthy controls and CKD patients. We observed a higher level of Lars2 in CKD samples compared with baseline group, with marginal significance (Baseline: 2.53; CKD: 2.66; p=0.067). **c.** LARS2 protein levels in WT and *Rtn3*-null primary cultured mice PT epithelial cells under control and hypoxic reperfusion conditions. LARS2 expression is elevated in *Rtn3*-null PT cells compared to WT cells, under both conditions.


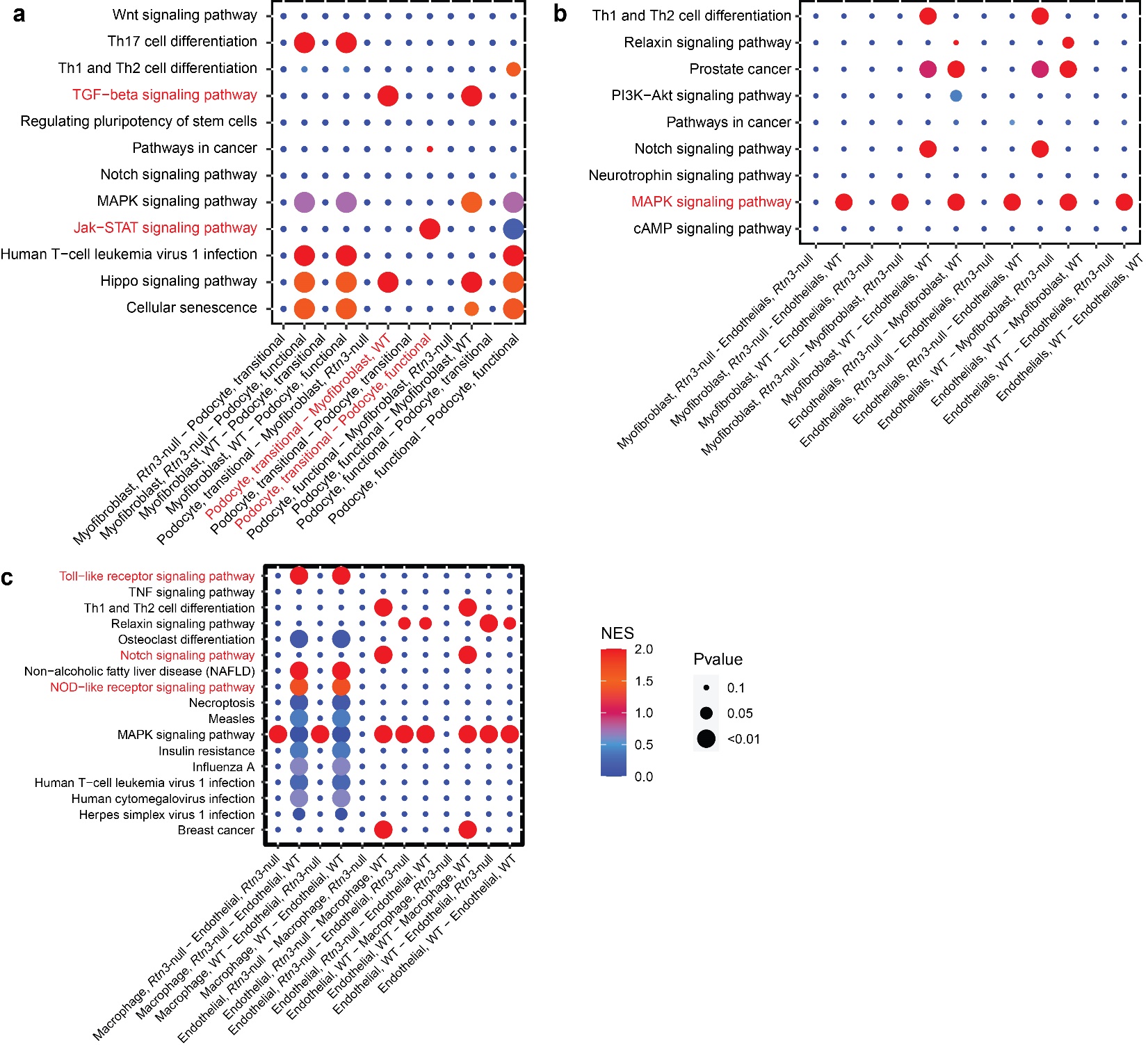


**Supplementary Figure 7. The impacts of *Rtn3*-null on cell-cell communication pathways.**

Dot plots showing the detailed impacts of *Rtn3*-null on the cell-cell communication pathways. **a**, MFs to PDs; **b**, MFs to ENs; **c**, ENs to MCs. PDs, Podocytes; ENs, Endothelial Cells; MCs, Macrophages; WT, Wild Type.


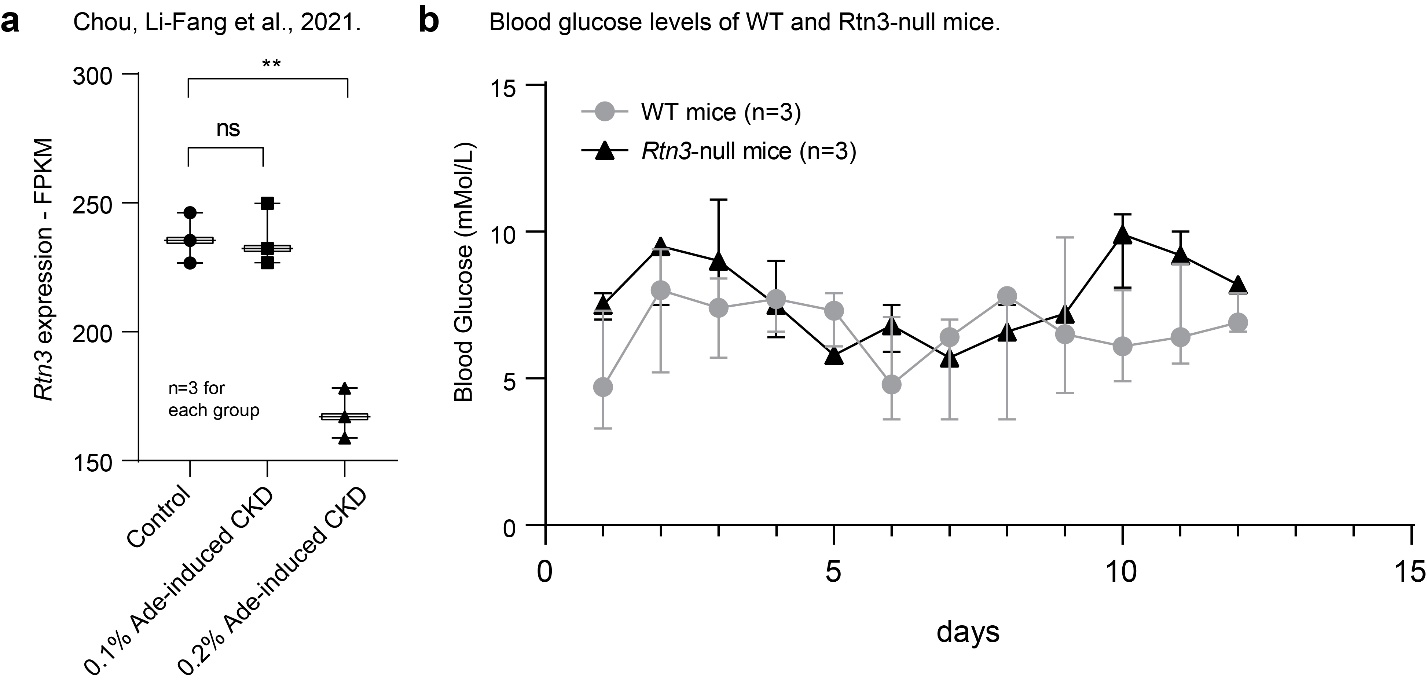


**Supplementary Figure 8. *Rtn3* and other impact factors.**

**a**, *Rtn3* expression levels in adenine-induced CKD mice; **b**, Blood glucose levels of WT and *Rtn3*-null mice.
